# Transcriptome Profiling of Porcine Naïve, Intermediate and Terminally Differentiated CD8^+^ T Cells

**DOI:** 10.3389/fimmu.2022.849922

**Published:** 2022-02-21

**Authors:** Emil Lagumdzic, Clara Pernold, Marta Viano, Simone Olgiati, Michael W. Schmitt, Kerstin H. Mair, Armin Saalmüller

**Affiliations:** ^1^ Department of Pathobiology, Institute of Immunology, University of Veterinary Medicine, Vienna, Austria; ^2^ Istituto di Ricerche Biomediche “A. Marxer” RBM S.p.A., Torino, Italy; ^3^ Merck Healthcare KGaA, Chemical & Preclinical Safety, Darmstadt, Germany; ^4^ Christian Doppler Laboratory for Optimized Prediction of Vaccination Success in Pigs, Department of Pathobiology, Institute of Immunology, University of Veterinary Medicine, Vienna, Austria

**Keywords:** CD8+ T cells, RNA-Seq, transcriptome, swine, T-cell differentiation

## Abstract

The pig has the potential to become a leading research model for human diseases, pharmacological and transplantation studies. Since there are many similarities between humans and pigs, especially concerning anatomy, physiology and metabolism, there is necessity for a better understanding of the porcine immune system. In adaptive immunity, cytotoxic T lymphocytes (CTLs) are essential for host defense. However, most data on CTLs come from studies in mice, non-human primates and humans, while detailed information about porcine CD8^+^ CTLs is still sparse. Aim of this study was to analyze transcriptomes of three subsets of porcine CD8β^+^ T-cell subsets by using next-generation sequencing technology. Specifically, we described transcriptional profiles of subsets defined by their CD11a/CD27 expression pattern, postulated as naïve (CD8β^+^CD27^+^CD11a^low^), intermediate differentiated (CD8β^+^CD27^dim^CD11a^+^), and terminally differentiated cells (CD8β^+^CD27^-^CD11a^high^). Cells were analyzed in *ex vivo* condition as well as upon *in vitro* stimulation with concanavalin A (ConA) and PMA/ionomycin. Our analyses show that the highest number of differentially expressed genes was identified between naïve and terminally differentiated CD8^+^ T-cell subsets, underlining their difference in gene expression signature and respective differentiation stages. Moreover, genes related to early (*IL7-R*, *CCR7*, *SELL*, *TCF7, LEF1*, *BACH2*, *SATB1*, *ZEB1* and *BCL2*) and late (*KLRG1*, *TBX21*, *PRDM1*, *CX3CR1, ZEB2*, *ZNF683*, *BATF*, *EZH2* and *ID2*) stages of CD8^+^ T-cell differentiation were highly expressed in the naïve and terminally differentiated CD8^+^ T-cell subsets, respectively. Intermediate differentiated CD8^+^ T-cell subsets shared a more comparable gene expression profile associated with later stages of T-cell differentiation. Genes associated with cytolytic activity (*GNLY*, *PRF1*, *GZMB*, *FASL*, *IFNG* and *TNF*) were highly expressed in terminally and intermediate differentiated CD8^+^ T-cell subsets, while naïve CD8^+^ T cells lacked expression even after *in vitro* stimulation. Overall, PMA/ionomycin stimulation induced much stronger upregulation of genes compared to stimulation with ConA. Taken together, we provided comprehensive results showing transcriptional profiles of three differentiation stages of porcine CD8^+^ T-cell subsets. In addition, our study provides a powerful toolbox for the identification of candidate markers to characterize porcine immune cell subsets in more detail.

## Introduction

CD8^+^ T cells play a key role in immune responses against intracellular pathogens by killing infected cells. Previous studies also identified their involvement in the destruction of tumor cells whereby an increased number of CD8^+^ T cells in colorectal, ovarian and gastric cancer was associated with a better overall survival (1–3). Furthermore, activated CD8^+^ T cells are responsible for major histocompatibility complex class I (MHC I) mediated allograft rejection (4). CD8^+^ T cells recognize peptide antigens presented by MHC class I molecules with their T-cell receptors (TCRs) and due to their striking feature of killing infected cells they are designated as cytotoxic T lymphocytes (CTLs). Their cytolytic activity is mediated through the release of cytotoxic granules, containing perforin and granzymes or Fas/Fas-Ligand interaction, leading to apoptosis of the target cells. Second, CTLs also produce cytokines such as interferon-γ (IFN-γ) and tumor necrosis factor (TNF), which show antimicrobial and antitumor properties (5, 6). Conventionally, differentiation stages of CD8^+^ T cells in the murine immune system can be delineated by CD44 and CD62L surface markers. Naïve CD8^+^ T cells (T_n_) are defined as CD44^low^CD62L^high^ cells, whereas effector CD8^+^ T cells (T_eff_) show a CD44^high^CD62L^low^ phenotype. Based on CD127 and KLRG1 expression, effector CD8^+^ T cells can be further differentiated into short-lived effector cells (SLEC) and memory precursor effector cells (MPEC) showing CD127^-^KLRG1^+^ and CD127^+^KLRG1^-^ phenotypes, respectively (7, 8). Moreover, low expression of CD11a and high expression of CD27 is associated with T_n_, while T_eff_ show high expression of CD11a and low expression of CD27. Expression levels of CD11a enable the identification of antigen-experienced CD8^+^ T cells and correlates positively with cytolytic activity and SLEC generation, whereas its absence favors formation of MPEC (9, 10). Different populations of CD8^+^ memory T-cells can be identified by using CD44, CD62L, CD69, CXCR1 and CD49d markers. Bach2 has been identified as being a transcription factor expressed on T_n_, while T-bet, Id2 and Blimp-1 are found on more differentiated T cells such as T_eff_ (11, 12). In the human immune system differentiation stages of CD8^+^ T cells are described based on the expression of four main surface markers, namely: CD45RA, CD27, CD28 and CCR7. With the combination of those markers, CD8^+^ T cells can be divided into T_n_ cells (CD27^+^CD28^+^CCR7^+^CD45RA^+^), early differentiated cells (CD27^+^CD28^+^CCR7^-^CD45RA^-^), early-like cells (CD27^-^CD28^+^CCR7^-^CD45RA^-^), intermediately differentiated cells (CD27^+^CD28^-^CCR7^-^CD45RA^-^), T-effector RA^+^ cells (CD27^−^CD28^−^CCR7^−^CD45RA^+^), T-effector RA^-^ cells (CD27^−^CD28^−^CCR7^−^CD45RA^-^) and central memory T cells (CD27^+^CD28^+^CCR7^+^CD45RA^-^) (13–17). Although the human and murine immune systems share similarities with the porcine immune system, detailed information about the phenotype and the differentiation stages of porcine CD8^+^ T cells is still sparse (18). Over the years, one of the major drawbacks to further characterizing CD8^+^ T cells is the absence of specific monoclonal antibodies against the respective differentiation antigens. An initial study on cellular response of porcine virus-specific CTLs against classical swine fever virus (CSFV) infected cells described them as CD4^-^CD5^+^CD6^+^ MHC-I restricted T lymphocytes (19). In 1999 Saalmüller et al. described that CD4^-^CD5^+^CD6^+^ cells with high expression of CD8α represent porcine CTLs (20). A more recent study defined CD2^+^CD3^+^CD4^−^CD5^high^CD6^+^CD8α^high^CD8β^+^ cells, which were also capable of perforin production, as porcine CTLs (21, 22). Previous studies by our group showed that naïve CD8^+^ T cells express CD27 and are negative for perforin, whereas the phenotype of more differentiated CD8^+^ T-cell subsets correlates with the increase of perforin and the decrease of CD27 expression (23). In this study we followed this hypothesis that the gradual change of CD27 expression, from intermediate to negative, indicates the transition from early to late effector or memory CD8^+^ T cells. Furthermore, we included CD11a for the discrimination of porcine CD8^+^ T-cell subsets, based on literature on CTL differentiation in mice (9, 10, 13). To confirm our hypothesis, we combined surface-antigen based cell sorting with transcriptome analysis of the respective subpopulations by using next-generation sequencing (NGS) technologies. We investigated three CD8^+^ T-cell subsets considered as the differentiation stages of naïve (CD8β^+^CD27^+^CD11a^low^), intermediate differentiated (CD8β^+^CD27^dim^CD11a^+^), and terminally differentiated cells (CD8β^+^CD27^-^CD11a^high^). So far, most of the transcriptomic studies in swine have addressed gene expression changes in peripheral blood mononuclear cells (PBMCs) only, i.e. upon vaccination or infection and our knowledge of the transcriptome profile of porcine CD8^+^ T-cells is largely based on limited data (24–26). To gain deeper insight into the differentiation of the CD8^+^ T cells we examined besides the direct *ex vivo* analyses the transcriptome changes after stimulation with different *in vitro* stimuli. Here, we include extensive gene ontology (GO) enrichment and pathway analysis, providing more detailed information about the immunological roles and functions of genes specific for the differentiation stages of porcine CD8^+^ T-cell subsets. Therefore, this study is an important contribution to the further characterization of the immune system in swine - a species with the potential to become a highly relevant preclinical model for human diseases and pharmacological questions as well as for transplantation studies.

## Materials and Methods

### Animals and Cell Isolation

Blood samples from swine were obtained from a local abattoir. Prior to blood sampling, animals were anesthetized electrically and sacrificed by exsanguination in accordance with Austrian Animal Welfare Slaughter Regulation. PBMCs were isolated from fresh heparinized blood of six animals of approximately six months of age by density gradient centrifugation (Pancoll human, density: 1.077 g/ml, PAN-Biotech, Aidenbach, Germany; 30 min at 920 x g).

### Magnetic-Activated Cell Sorting (MACS)

CD8^+^ T cells were enriched by positive selection of CD8β-labeled PBMCs using magnetic-activated cell sorting (MACS, Miltenyi Biotec, Bergisch Gladbach, Germany). For enrichment of CD8β^+^ T cells, freshly isolated PBMCs (1 x 10^9^) were stained with an in-house produced primary monoclonal anti-CD8β antibody (clone PPT23, IgG1) for 20 min on ice. Subsequently, cells were washed once with MACS buffer [PBS w/o Ca/Mg + 2% (v/v) FCS (both Gibco™, Thermo Fisher Scientific) + 2mM EDTA (Carl Roth)], resuspended in 1,5 mL MACS buffer and incubated with magnetically labeled secondary antibody (rat-anti mouse IgG1, Miltenyi Biotec) for 30 min on ice. After a further washing step, cells were resuspended in 3 mL MACS buffer and loaded on pre-wetted LS columns (Miltenyi Biotec). The columns were applied to a magnetic field and unlabeled cells were removed by extensive washing. For final elution of the positive fraction, columns were removed from the magnetic field and CD8β^+^ T cells were eluted in 5 mL MACS buffer. Finally, sorted cells were resuspended in cold culture medium (RPMI 1640 + 100 IU/mL penicillin + 0.1 mg/mL streptomycin (all PAN Biotech) + 10% (v/v) FCS), centrifuged and counted with a Cell Counter (XP-300 Hematology Analyzer, Sysmex Europe GmbH, Norderstedt). Purity of the positively sorted cells was over 90% (FACSCanto™II, BD Biosciences, San Jose, CA, USA).

### Fluorescence-Activated Cell Sorting (FACS)

In order to further separate MACS-enriched CD8β^+^ cells into subpopulations, CD8β^+^ cells were FACS sorted based on surface expression of CD27 and CD11a (**Supplementary Figure S1**).

Upon magnetic-activated cell sorting, CD8β^+^ cells were washed once with FACS buffer (RPMI 1640 + 100 IU/mL penicillin + 0.1 mg/mL streptomycin + 5% FCS + 5% porcine plasma (in-house preparation) + 2 mM EDTA) and then labeled with a goat anti-mouse IgG1-PE secondary antibody to stain residual CD8β^+^ cells (Southern Biotech, Birmingham, AL, USA).

Free binding sites of the PE-labeled antibody were blocked with whole mouse IgG molecules (2 μg per sample, ChromPure, Jackson ImmunoResearch, West Grove, PA, USA). Afterwards, cells were incubated with directly labeled primary antibodies: CD27-Alexa647 (b30c7, mouse IgG1, in-house preparation and labeling with Alexa Fluor-647 Protein Labeling Kit, Thermo Fisher Scientific) and CD11a-FITC (BL1H8, mouse IgG2b, BioRad, Hercules, CA, USA).

Cell sorting was performed on a FACSAria (BD Biosciences) and CD8^+^ T-cell subsets were defined as follows: naïve (CD8β^+^CD27^+^CD11a^low^), intermediate differentiated (CD8β^+^CD27^dim^CD11a^+^), and terminally differentiated cells (CD8β^+^CD27^-^CD11a^high^). Subsets were sorted with an average purity greater than 96%.

### *In Vitro* Stimulation

To identify transcriptomic differences between the CD8^+^ T-cell subsets as well as between *ex vivo* and stimulated cells within the same CD8^+^ T-cell subset, cells from each sorted subpopulations with at least 5 x 10^5^ sorted cells were cultivated at 37°C and 5% CO_2_ under following conditions: (i) 16 hours, unstimulated in culture medium, (ii) cultivation in culture medium for 14 hours followed by stimulation for two hours with phorbol 12-myristate 13-acetate (PMA, 50 ng/mL, Sigma-Aldrich, Schnelldorf, Germany) and ionomycin (500 ng/mL, Sigma-Aldrich), (iii) stimulated with concanavalin A (ConA) (5 μg/mL, Amersham Biosciences, Uppsala, Sweden) for 16 hours. Both stimulation protocols are established in our laboratory and used as high controls for proliferation experiments and cytokine induction in ELISpot assays (ConA) and as positive control for intracellular cytokine staining in flow cytometry (PMA/ionomycin). Furthermore, each CD8^+^ T-cell subset with 5 x 10^5^ was used immediately after sorting for RNA isolation without any further cell culture (*ex vivo*). Altogether four different conditions for each CTL subset were applied: cultivation in medium, stimulation with PMA/ionomycin or ConA and *ex vivo* isolation. Therefore, 72 samples (3 subsets x 4 conditions x 6 animals) were generated.

### RNA Extraction, Library Preparation and Sequencing

Total RNA was isolated from the samples mentioned above using RNeasy Mini Kit with on-column DNase treatment using the RNAse-Free DNase Set (both Qiagen, Hilden, Germany), following manufacturer’s protocol. Quantification and quality control of isolated RNA were assessed with both Qubit 3.0 fluorometer (RNA HS assay kit, ThermoFisher, Massachusetts, MA, USA) and Agilent 2100 Bioanalyzer (Agilent RNA 6000 Pico Kit, Agilent Technologies, Palo Alto, CA, USA). Samples with both a final yield comprised between 0.03 – 1.25 ng/µl and a RIN of 9 were prepared for sequencing with the SMARTer Stranded Total RNA-Seq v2 – Pico Input Mammalian Kit (Takara Bio Inc., Shiga, Japan). Fully automated library preparation was performed on a Microlab Star Hamilton robotic station (Hamilton Company, Reno, NV, USA). Briefly, 8 µl per sample were used for the cDNA synthesis *via* the SMART^®^ technology (SMART technology, Clontech, USA). Thereafter, each sample was amplified to generate Illumina-compatible libraries according to the manufacturer’s guidance. Libraries were validated using the Agilent 2100 Bioanalyzer (Agilent High Sensitivity DNA Kit, Agilent Technologies, Palo Alto, CA) and the Qubit 3.0 fluorometer (DNA HS assay kit, ThermoFisher, Massachusetts, MA, USA). Libraries were paired-end sequenced on two SP flow cell on NovaSeq 6000 system (Illumina Inc., San Diego, CA, USA).

### Mapping and Differential Gene Expression Analysis (DGE)

Standard raw sequencing data in BCL format was converted to FASTQ files using the software bcl2fastq v2.19.1.403. After importing the FASTQ files into CLC Genomics Workbench 21.0.3 (Qiagen, Aarhus, Denmark), the reads were adapter- and quality trimmed. Prior to mapping, sequence reads were trimmed using quality score (Phred score ≤ 25) and with maximum number of 2 ambiguous nucleotides allowed. Next, the adapter sequences were trimmed off according to the Illumina Adapter List. Reads shorter than 35 and longer than 75 nucleotides were discarded.

The filtered reads were mapped to the Sus scrofa 11.1 reference genome from NCBI database (GCA_000003025.6) using default parameters of CLC Genomics RNA-Seq Analysis tool (mismatch cost = 2, insertion cost = 3, deletion cost = 3, length fraction = 0.8 and similarity fraction = 0.8). For principal component analysis (PCA), mapped reads were TMM normalized, log CPM values calculated and Z-normalization performed. For the *ex vivo* condition, differential gene expression test for differences between all pairs of CD8^+^ T-cell subsets using Wald test was performed. Therefore, three pairwise comparisons were made: (i) naïve vs. terminally differentiated, (ii) intermediate vs. terminally differentiated, and (iii) naïve vs. intermediate differentiated. To assess the effect of stimulation on gene expression profiles of CD8^+^ T-cell subsets, Wald test with medium condition as control group was used. Correspondingly, that yielded two pairwise comparisons for each CD8^+^ T-cell subset: (i) ConA stimulation vs. medium and (ii) PMA/ionomycin stimulation vs. medium. As criteria to define differentially expressed genes (DEGs), fold-change > |2|, maximum of the average reads per kilobase per million mapped reads (RPKM’s) > 2 and a false discovery rate corrected p-value < 0.01 (FDR) were used. Venn diagram and heat map visualization of DEGs were constructed using ggvenn and pheatmap packages in R software version 4.0.2 (R Core Team, GNU General Public License). Bar charts were visualized with Tableau Desktop 2020.3 (Tableau Software Inc.).

### Gene Ontology Enrichment and Pathway Analysis

For DEGs, gene ontology (GO) and enrichment analysis for immune system processes were executed using the ClueGO v.2.5.8 plug-in in the bioinformatic software Cytoscape 3.8.2. version (https://cytoscape.org). The analysis was performed for upregulated genes between CD8^+^ T-cell subsets and based on GO data for Sus scrofa. Following cut-off thresholds were set: at least 3 genes per GO term, two-sided hypergeometric statistical testing corrected with the Bonferroni step-down method (p < 0.05) and a Kappa score of 0.4. Moreover, organism-specific pathway analysis of DEGs were constructed by using KEGG mapper based on Kyoto Encyclopedia of Genes and Genomes (KEGG) pathway database with KEGG Orthology (KO) assignment.

## Results

### Gene Expression Profiles of *Ex Vivo* Sorted CD8^+^ T-Cell Subsets

Based on our hypothesis that within the CD8β^+^ T-cell subpopulation three subsets with distinct differentiation stages can be defined, we analyzed the presumable naïve (T_n_; CD8β^+^CD27^+^CD11a^low^), intermediate differentiated (T_inter_; CD8β^+^CD27^dim^CD11a^+^), and terminally differentiated cells (T_term_; CD8β^+^CD27^-^CD11a^high^).

**Figure 1:**
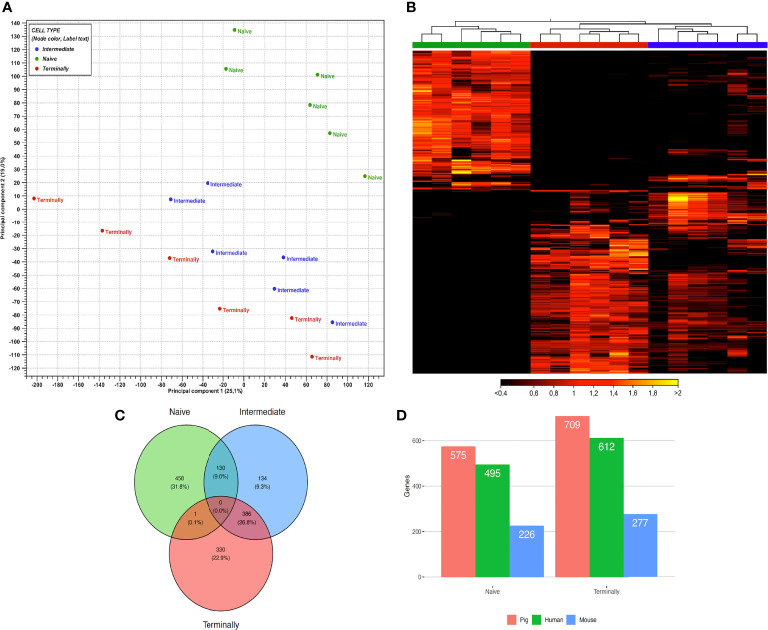
Gene expression profiles of *ex vivo* sorted CD8^+^ T-cell subsets. **(A)** PCA plot of expression data derived from 18 *ex vivo* CD8^+^ T-cell subsets of six animals. Colors indicate three CD8^+^ T-cell subsets: green, naïve CD8^+^ T cells; blue, intermediate differentiated CD8^+^ T cells; red, terminally differentiated CD8^+^ T cells. PC1 explains 25.1% and PC2 explains 19% of the observed variance in data. **(B)** Heat map with hierarchical clustering of 1439 selected genes between *ex vivo* CD8^+^ T-cell subsets. Figure illustrates clustering of three CD8^+^ T-cell subsets based on gene expression values. Rows represent genes and columns samples, with yellow indicating high and black low expression. The dendrogram on the top indicates the correlation between samples. Colors underneath the dendrogram represent three CD8^+^ T-cell subsets, namely: naïve (green), terminally (red) and intermediate (blue). **(C)** Venn diagram showing the overlap of 1439 DEGs between *ex vivo* CD8^+^ T-cell subsets. Colors indicate three main CD8^+^ T-cell subsets: green, naïve CD8^+^ T cells; blue, intermediate differentiated CD8^+^ T cells; red, terminally differentiated CD8^+^ T cells. **(D)** Orthologous genes from DEGs in porcine naïve and terminally differentiated CD8^+^ T-cell subsets compared to human and mouse.

In total 3.59 billion paired-end reads were generated by sequencing 72 libraries. Overall, the percentage of mapping reads to the reference genome was between 90.44% and 94.87% (mean = 93.1%) with approximately 50 million paired-end reads per sample. PCA of gene expression data from all *ex vivo* CD8^+^ T-cell subsets revealed distinguishable differences between CTL subsets as PCA plot clustered data into three distinct groups (**Figure 1A**).

For hierarchical cluster analysis, we selected 1439 genes, which were significantly expressed in at least one pairwise comparison between CD8^+^ T-cell subsets (as defined in the Methods section). Afterwards, a heat map based on their gene expression values was generated. Notably, the hierarchical clustering of selected genes identified three well-defined groups of samples. The first contained all T_n_ samples, the second all T_term_ samples and the third all T_inter_ samples (**Figure 1B**). Genes highly expressed in T_n_ were downregulated in T_term_ and vice versa. This clear separation regarding gene expression could indicate transcriptional switch that CD8^+^ T cells undergo while differentiating from naïve to terminally differentiated CD8^+^ T cells. In comparison to T_n_ and T_term_, T_inter_ showed upregulation of genes expressed in both groups. However, Venn diagram analysis showed that T_inter_ and T_term_ share more DEGs (n=386) than T_inter_ and T_n_ (n=130) (**Figure 1C**). In contrast, only one upregulated DEGs was shared between the T_n_ and T_term_ when compared to T_inter_. Next, using Wald test for pairwise comparison, 575 and 709 DEGs were identified as upregulated in T_n_ and T_term_, respectively (**Supplementary Table S1**). The number of upregulated DEGs was smaller in T_inter_ vs. T_term_ comparison than T_n_ vs. T_inter_ comparison. A higher number of upregulated DEGs (n = 492) was observed in T_inter_ compared to T_n_ (n = 215) CD8^+^ T cells. Also, higher numbers of upregulated DEGs were discovered in T_inter_ (n = 208) than in T_term_ (n = 132). To obtain further information about each stage of CD8 T-cell differentiation, gene expression profiles were compared between T_n_, T_inter_ and T_term_. We found that genes related to early stages of CD8 T-cell differentiation were highly expressed in the T_n_ (**Table 1**). Expression of several genes encoding transcription factors associated with naïve lymphocytes (27), including *LEF1*, *BACH2*, *TCF7* (TCF1), *SATB1*, *ZEB1* and *BCL2* were markedly increased in the T_n_. In contrast, genes encoding transcription factors associated with terminally differentiated effector cells, such as *TBX21* (T-bet), *PRDM1* (Blimp-1), *ZEB2*, *ZNF683* (Hobit), *BATF*, *EZH2* and *ID2* were highly upregulated in the T_term_.

**Table 1:** Selected differentially expressed genes between *ex vivo* T_n_, T_inter_ and T_term_.

	T_n_ vs. T_term_			T_n_ vs. T_inter_			T_inter_ vs. T_term_		
Gene name	Fold change		p-value	Fold change		p-value	Fold change		p-value
CCR7	535.18	–	4.2E-118	5.73	–	8.0E-17	93.32	–	5.8E-60
LEF1	95.23	–	8.7E-104	6.04	–	9.7E-17	15.77	–	3.4E-37
MYB	76.28	–	1.1E-72	11.64	–	1.9E-35	–	–	–
SELL	62.31	–	7.4E-35	4.97	–	1.1E-05	12.54	–	1.5E-12
IL7R	40.14	–	8.6E-72	2.38	–	1.3E-04	16.89	–	4.5E-41
CD27	29.30	–	6.5E-54	2.20	–	8.1E-04	13.34	–	9.4E-31
TCF7	26.40	–	4.0E-79	2.17	–	6.7E-05	12.14	–	6.9E-45
ZEB1	18.13	–	7.3E-78	2.23	–	3.2E-07	8.11	–	2.5E-39
MYC	14.95	–	5.0E-51	2.81	–	8.7E-08	5.32	–	8.9E-19
KLF9	12.43	–	3.9E-12	3.53	–	9.2E-04	–	–	–
CD28	12.12	–	9.1E-21	–	–	–	12.30	–	5.2E-20
CCR9	9.82	–	1.6E-16	2.47	–	2.8E-03	3.98	–	1.0E-05
BACH2	8.44	–	2.6E-33	3.80	–	3.0E-13	–	–	–
BCL2	8.13	–	7.8E-18	2.65	–	3.0E-04	3.06	–	5.7E-05
SATB1	8.04	–	1.7E-26	3.00	–	1.3E-07	2.68	–	9.4E-06
HIF1A	5.52	–	2.0E-21	–	–	–	3.79	–	2.0E-12
TNFRSF25	4.24	–	1.3E-10	–	–	–	5.68	–	7.7E-10
S1PR1	4.15	–	2.7E-23	–	–	–	3.23	–	4.6E-15
SOCS3	3.69	–	1.3E-03	–	–	–	8.91	–	3.5E-08
FOXP1	3.01	–	9.2E-19	2.35	–	3.6E-11	–	–	–
GNLY	–	1457.47	1.1E-230	–	372.87	1.1E-151	–	3.91	8.3E-11
ADGRG1	–	996.20	6,5E-221	–	285.90	1,4E-147	–	3.48	1,7E-09
CX3CR1	–	582.11	5,2E-46	–	193.70	4,3E-31	–	–	–
ZEB2	–	458.99	1,2E-239	–	143.51	3,0E-156	–	3.20	2,6E-15
S1PR5	–	377.46	3,9E-100	–	110.16	5,6E-62	–	3.43	1,0E-08
ITGAM	–	207.10	4,9E-48	–	200.19	9,5E-47	–	–	–
PRDM1	–	173.10	5,9E-114	–	73.37	2,9E-78	–	2.36	4,5E-04
GZMB	–	120.37	9,2E-65	–	58.46	4,5E-46	–	–	–
FASLG	–	118.25	1,2E-25	–	61.36	1,1E-18	–	–	–
CCL5	–	103.94	1,0E-67	–	72.50	4,2E-57	–	–	–
KLRD1	–	99.05	9,9E-49	–	–	–	–	2.56	2,7E-03
TBX21	–	87.10	1,3E-60	–	38.71	4,0E-40	–	–	–
KLRG1	–	77.32	8,5E-77	–	23.61	1,6E-39	–	3.28	3,8E-12
IFNG	–	41.96	4,3E-19	–	33.40	2,0E-16	–	–	–
KLRK1	–	37.58	4,7E-36	–	28.56	2,2E-30	–	–	–
GZMA2	–	34.53	1,3E-34	–	50.00	1,4E-41	–	–	–
SLC1A5	–	33.96	1,9E-34	–	21.16	2,7E-25	–	–	–
LGALS1	–	32.25	1,3E-38	–	25.96	2,3E-33	–	–	–
TNFAIP2	–	30.51	1,3E-23	–	10.66	2,9E-11	–	–	–
IL2RB	–	23.10	1,5E-52	–	15.97	2,1E-40	–	–	–
CCR5	–	20.13	3,6E-20	–	27.82	3,4E-24	–	–	–
ZNF683	–	19.64	3,5E-46	–	12.24	2,9E-32	–	–	–
BATF	–	14.91	2,5E-23	–	10.04	1,1E-16	–	–	–
TNF	–	14.20	2,6E-16	–	–	–	–	2.74	4,7E-03
TNFSF12	–	13.51	5,1E-42	–	7.50	1,4E-24	–	–	–
CCL4	–	11.64	2,7E-09	–	15.43	4,4E-11	–	–	–
PRF1	–	9.63	2,9E-32	–	5.39	1,2E-17	–	–	–
PDCD1	–	9.23	5,2E-13	–	8.01	3,9E-11	–	–	–
ANXA2	–	9.14	1,1E-22	–	9.13	3,2E-22	–	–	–
ITGAL	–	7.54	1,4E-29	–	4.31	2,5E-15	–	–	–
IL12RB2	–	7.22	8,3E-24	–	4.49	1,5E-13	–	–	–
MKI67	–	6.80	2,7E-11	–	19.78	3,8E-26	2.91	–	1,5E-03
TNFRSF1B	–	6.23	5,1E-19	–	5.38	8,6E-16	–	–	–
FAS	–	5.41	7,1E-28	–	5.73	2,7E-29	–	–	–
NFKBIE	–	4.52	6,3E-12	–	3.23	3,9E-07	–	–	–
IRF8	–	4.47	1,1E-08	–	6.08	5,3E-12	–	–	–
ITGB2	–	4.39	9,8E-27	–	2.73	3,0E-12	–	–	–
TNFRSF1A	–	4.24	4,0E-12	–	2.28	3,2E-04	–	–	–
RUNX3	–	4.05	1,4E-22	–	2.39	7,4E-09	–	–	–
SOCS1	–	3.66	8,5E-19	–	–	–	–	2.02	1,6E-05
ID2	–	3.62	2,2E-14	–	–	–	–	2.08	1,4E-04
TNFSF10	–	3.46	9,3E-10	–	2.51	2,1E-05	–	–	–
EZH2	–	2.92	1,0E-09	–	3.80	2,4E-14	–	–	–
ARNTL	–	2.88	6,3E-15	–	2.05	6,9E-07	–	–	–
GZMM	–	2.82	3,4E-11-	–	–	–	–	–	–
IL12RB1	–	2.77	6,6E-06	–	2.39	2,5E-04	–	–	–
ITGA4	–	2.58	4,9E-12	–	2.24	1,3E-08	–	–	–
SOCS7	–	2.29	6,5E-14	–	–	–	–	–	–
STAT4	–	2.28	7,1E-12	–	–	–	–	–	–
IL2RG	–	2.15	7,0E-07	–	2.36	3,7E-08	–	–	–

Furthermore, T_term_ showed high expression of several genes involved in cell adhesion and migration including *CX3CR1*, *CCR5*, *CCL4* and *CCL5*. Moreover, higher expression of adhesion genes *ITGAM* (CD11b) and *ITGAL* (CD11a) (28) was observed among T_term_ compared with T_n_ and T_inter_. Expression of *ITGA4* (CD49d), which together with CD44 is expressed in effector T cells and effector memory T cells (13), was upregulated in T_inter_ and T_term_. In addition, expression of *CD44* was increased in both T_inter_ and T_term_ but not in the T_n_ (**Supplementary Table S1**). Conversely, genes encoding lymph node homing receptor molecules such as *CCR7*, *SELL* (CD62L) and *CCR9* were highly upregulated in the T_n_. Sphingosine-1-Phosphate Receptor 1 (*S1PR1*), important for lymphocyte trafficking and upregulated in human naïve T cells (29), was also increased in the porcine T_n_. Also, T_n_ showed high expression of genes encoding CD27 and CD28 molecules, the former in accordance with cell surface expression used for the sorting strategy.

We observed that several genes involved in T-cell effector functions and cytolytic killing, including *GNLY* (Granulysin), *PRF1* (Perforin), *GZMB* (Granzyme B), *FAS*, *FASL*, *IFNG* and *TNF*, were highly increased in T_term_ in comparison to T_n_ or T_inter_. Notably, T_term_ expressed the *GNLY* 1457-fold higher in comparison to T_n_. Moreover, T_term_ showed high expression of *KLRG1*, *KLRD1* and *KLRK1*, whereas T_n_ displayed high mRNA levels of *IL-7R* (CD127). In mouse a selective expression of *IL-7R* (CD127) is used for the discrimination between MPEC and SLEC, with the high expression specific for MPEC (8). In addition to the high expression of *IL-7R*, human MPEC show low expression of *KLRG1*, while SLEC show upregulation of *KLRG1* and low expression of *IL7R* (13).

We found higher expression of co-inhibitory molecule *PDCD1* (PD-1) in T_inter_ and T_term_ when compared to the T_n_. Previous research suggests that high expression of *PDCD1* (PD-1) is specific for SLEC formation, whereas low *PDCD1* expression contributes to the T effector memory generation (30). Several genes encoding cytokine receptors associated with effector T cells were increased in the T_term_, including *IL2RB* (CD122), *IL2RG* (CD132), *IL12RB1* and *IL12RB2*. In comparison to the T_n_, T_inter_ and T_term_ showed high expression of *IRF8*, which supports the transition from naïve to effector CD8^+^ T cells in independent matter to T-bet and Eomes (31). Furthermore, upregulation in transcript levels of *ITGB2* (CD18) and *ANXA2*, known to be increased in CD8^+^ effector T cells (32), as well as *LGALS1*, which is expressed only on activated CD8^+^ effector T cells but not resting CD8^+^ T cells (33), were observed in T_inter_ and T_term_. Additionally, genes strongly linked to cytotoxic T cells such as *S1PR5* and *ADGRG1* were substantially upregulated in the T_term_. By contrast, T_n_ showed high expression of genes, which enforce quiescence state of naïve T cells (*MYB*, *FOXP1*, *KLF9* and *SOCS3*). In comparison to T_n_, we found other members of SOCS family, namely SOCS1 and SOCS7, highly expressed in T_term_. Furthermore, expression of *MKI67*, encoding proliferation marker Ki-67, was upregulated in T_inter_ and T_term_. Both *TNFRSF1A* (TNFR1) and *TNFRSF1B* (TNFR2) were upregulated in T_inter_ and T_term_. While transcripts of *TNFSF12* (TWEAK) and *TNFSF10* (TRAIL) were upregulated in T_inter_ and T_term_, expression of costimulatory *TNFRSF25* (DR3) was highly induced in the T_n_. Only T_term_ expressed high levels of *GZMM* and *STAT4*, on the other hand T_inter_ showed upregulation of *GZMA2*. Notably, the expression of *RUNX3*, which is important for the acquisition and maintenance of cytolytic functions of CD8^+^ effector T cells (34), was upregulated in T_inter_ and T_term_. Furthermore, T_inter_ and T_term_ showed increased levels of *TNFAIP2* and *NFKBIE*. Regarding genes involved in metabolism, we observed high expression of *ARNTL* and *SLC1A5* in more differentiated CTL subsets, whereas expression of *HIF1A* was upregulated in the T_n_. Interestingly, when compared to T_n_ and T_term_, T_inter_ shared a more comparable gene expression profile associated with later stages of T-cell differentiation. In particular, most of genes highly expressed in T_term_ were also upregulated in T_inter_. However, the difference in expression of genes related to early stages of T-cell differentiation was substantially smaller between T_n_ and T_inter_ than T_n_ and T_term_. Also, those genes were higher expressed in T_inter_ than T_term_.

### Identification of Swine Orthologous Genes in Human and Mice

For better understanding of the relationship between porcine, human and mouse CD8^+^ T cells we assessed the orthology of their genes expressed in CD8^+^ T cells in corresponding subsets publicly available on GEO Data sets (NCBI) under GDS3834 and GDS592. Here, we have focused on the analysis of DEGs in T_n_ and T_term_, which cover the vast majority of DEGs generated from porcine CD8^+^ T-cell subsets. When compared to DEGs in porcine T_n_, we found 495 (86.1%) orthologs in human and 226 (39.3%) in mouse data set. In case of T_term_, out of 709 DEGs, 612 (86.3%) were recorded in human, and 277 (39.1%) in mouse data set (**Figure 1D**).

### Gene Signature of *In Vitro* Stimulated CD8^+^ T-Cell Subsets

**Figure 2:**
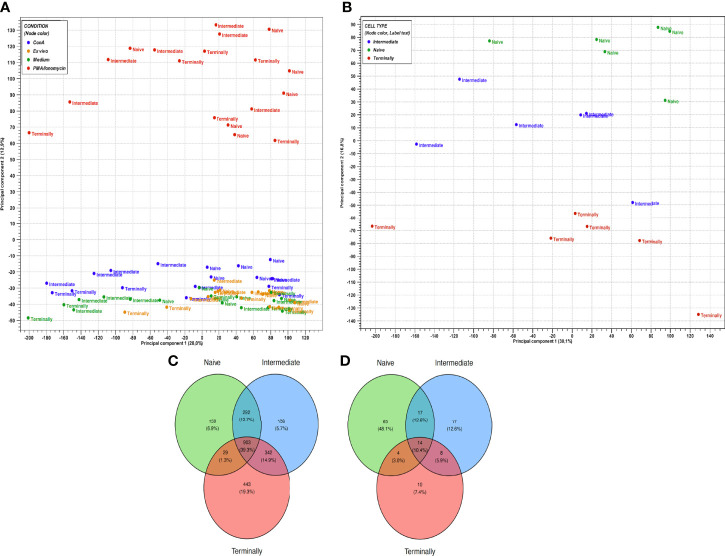
Gene expression profiles of *in vitro* stimulated CD8^+^ T-cell subsets. **(A)** PCA plot of expression data derived from 72 CD8^+^ T-cell subsets of six animals. Colors indicate four conditions: blue, ConA stimulation; orange, *ex vivo* condition; green, medium control; red, PMA/ionomycin stimulation. Cross, X symbol and square shape represent naïve, intermediate and terminally differentiated CD8^+^ T-cell subsets, respectively. PC1 explains 28% and PC2 explains 12.9% of the observed variance in data. **(B)** PCA plot of expression data derived from 18 PMA/ionomycin-stimulated CD8^+^ T-cell subsets of six animals. Colors indicate three CD8^+^ T-cell subsets: green, naïve CD8^+^ T cells; blue, intermediate differentiated CD8^+^ T cells; red, terminally differentiated CD8^+^ T cells. PC1 explains 30.1% and PC2 explains 16.8% of the observed variance in data. **(C)** Venn diagram showing the overlap of DEGs between PMA/ionomycin-stimulated CD8^+^ T-cell subsets. **(D)** Venn diagram showing the overlap of DEGs between ConA-stimulated CD8^+^ T-cell subsets.

In order to further highlight the heterogeneity in gene expression between CD8^+^ T-cell subsets, cells were analyzed upon stimulation with ConA and PMA/ionomycin and compared to cells cultured in medium control. Overall, a substantially higher number of upregulated DEGs in all three CD8^+^ T-cell subsets was observed in response to PMA/ionomycin compared to ConA stimulation. Additionally, gene expressions of all PMA/ionomycin-stimulated CD8^+^ T-cell subsets are clearly distinct from all other CD8^+^ T-cell subsets as showed in PCA plot (**Figure 2A**). Further investigation of CD8^+^ T-cell subsets stimulated with PMA/ionomycin revealed the highest number of upregulated DEGs in the T_term_ (1717), followed by the T_inter_ (1667) and the T_n_ (1383). Conversely, in CD8^+^ T-cell subsets stimulated with ConA, the highest number of DEGs was found in the T_n_ (100), followed by the T_inter_ (35) and the T_term_ (36) (**Supplementary Table S1**). In order to obtain a more detailed view upon PMA/ionomycin stimulation, PCA was performed additionally only on PMA/ionomycin-stimulated CD8^+^ T-cell subsets (**Figure 2B**). Interestingly, CD8^+^ T-cell subsets clustering is unaltered to PMA/ionomycin stimulation, resulting again in the three distinct groups of T_n_, T_inter_ and T_term_. Despite this separate clustering, Venn diagram analysis revealed high number of DEGs shared between PMA/ionomycin-stimulated CD8^+^ T-cell subsets (903), indicating that all three subsets acquire more similar cell properties following PMA/ionomycin stimulation. In case of ConA-stimulated CD8^+^ T-cell subsets, we found much smaller number of shared DEGs (**Figure 2C, D**).

**Figure 3:**
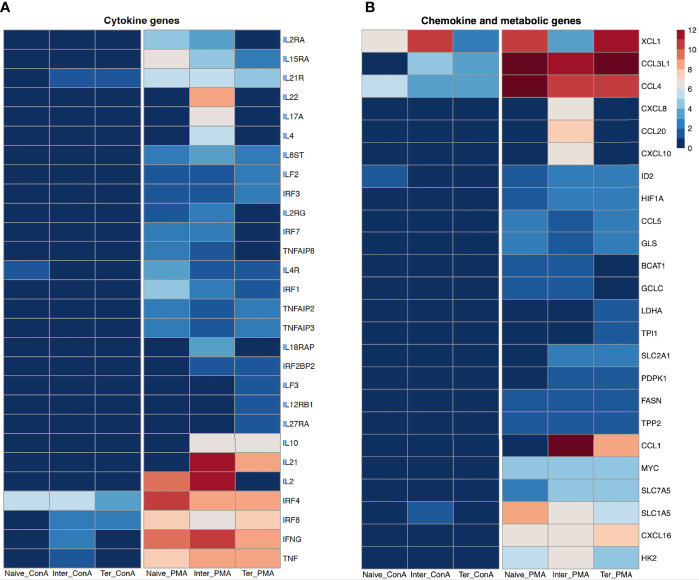
Transcription profiles of naïve, intermediate and terminally differentiated CD8^+^ T-cell subsets. Data derived from 18 ConA and 18 PMA/ionomycin-stimulated CD8^+^ T-cell subsetsof six animals. As criteria to define DEGs, fold-change > |2| compared to medium control, maximum of the average RPKM’s > 2 and a false discovery rate corrected p-value < 0.01 (FDR) were used. **(A)** Expression of cytokine genes as log2 fold-change between ConA- and PMA/ionomycin-stimulated CD8^+^ T-cell subsets and their unstimulated control. **(B)** Expression of chemokine and metabolic genes as log2 fold-change between ConA- and PMA/ionomycin-stimulated CD8^+^ T-cell subsets and their unstimulated control.

Several genes encoding cytokines involved in T-cell response were differentially expressed between CD8^+^ T-cell subsets (**Figure 3A**). Looking at the expression of *IFNG* (IFN-γ) and *TNF*, we observed overexpression in all three CD8^+^ T-cell subsets following PMA/ionomycin stimulation, but only moderate expression in T_inter_ with ConA stimulation. Porcine T_n_ and T_inter_ showed high expression of *IL2* and its receptor chains *IL2RA* (CD25) and *IL2RG* (CD132) as well as *IRF7* when stimulated with PMA/ionomycin. It was reported that IL2 and its receptor chains IL2RA (CD25) and IL2RG (CD132) are involved in terminal effector differentiation but also in memory development of CD8^+^ T cells (37). Moreover, expression of *IL4*, *IL17A*, *IL18RAP* and *IL22* was induced only in the T_inter_ stimulated with PMA/ionomycin. In contrary, *IL12RB1*, *IL27RA* and *ILF3* were only expressed by the T_term_. Nevertheless, both CD8^+^ T-cell subsets showed high expression of *IL10* and *IRF2BP2* after PMA/ionomycin stimulation. Although the expression of *IRF4* was upregulated in all three CD8^+^ T-cell subsets following PMA/ionomycin and ConA stimulations, the highest expression was induced by PMA/ionomycin-stimulated T_n_ followed by T_inter_ and T_term_. Consistently, studies in mice showed that IRF4 contributes to expansion and maintenance of effector functions of CTL as well as to memory formation of CTL (36). In case of *IRF8* transcript, we found similar expression between CD8^+^ T-cell subsets stimulated with PMA/ionomycin. In comparison, ConA stimulation induced a much smaller extent expression of *IRF8* in T_inter_ and T_term_. Expression of both genes, *IL6ST* and *ILF2* were similarly increased in all three CD8^+^ T-cell subsets upon PMA/ionomycin stimulation. Remarkably, the highest expression of *IL4R*, *IL15RA* and *IRF1* was recorded in the T_n_ followed by T_inter_ and T_term_. Of interest, we found three genes of TNF-induced proteins, namely *TNFAIP2*, *TNFAIP3* and *TNFAIP8* highly expressed in CD8^+^ T-cell subsets following PMA/ionomycin stimulation. Moreover, expression of *TNFAIP2* and *TNFAIP3*, both inhibiting canonical NF-kB signaling pathway and thus negatively effecting cytokine production, was highest in the T_n_ (38, 39). Apart from its aforementioned functions, *TNFAIP3*, also highly expressed on naïve T cells, restricts MAP kinases and CD8^+^ T cell proliferation (40).

Chemokines and chemokine receptors play a pivotal role in attracting and guiding the naïve and effector T cells to lymph nodes and sites of inflammation, respectively (41). Overall, in all three CD8^+^ T-cell subsets the PMA/ionomycin stimulation induced stronger expression of genes associated with chemokines than after ConA stimulation (**Figure 3B**). Expression of *CCL4* (MIP-1ß) and *XCL1* (ATAC/lymphotactin), the inflammatory chemokines secreted by activated CD8^+^ T cell (42), was induced in all three CD8^+^ T-cell subsets upon both stimulations, although with significantly higher increase in PMA/ionomycin-stimulated CD8^+^ T-cell subsets. All three PMA/ionomycin-stimulated CTL subsets showed similar expression of *CCL3L1*, while following ConA stimulation it was increased in T_inter_ and T_term_. Interestingly, only T_inter_ stimulated with PMA/ionomycin showed significant increase in *CCL20*, *CXCL8* and *CXCL10* expression, later known as one of interferon-inducible ligands of CXCR3 (43). The PMA/ionomycin stimulation induced also transcriptional upregulation of *CCL5* (RANTES) and *CXCL16* in all three CD8^+^ T-cell subsets. Furthermore, transcription of *CCL1* was increased in T_inter_ and T_term_ upon PMA/ionomycin stimulation.

Transition from naïve T cell to activated effector T cell is accompanied by metabolic adjustment necessary for specific cellular functions (44). Overall, the PMA/ionomycin stimulation induced stronger expression of genes linked to T-cell metabolism in comparison to the ConA stimulation. T_n_ and T_inter_ upregulated *BCAT1* and *GCLC* upon PMA/ionomycin stimulation, while T_term_ were enriched in transcripts for *LDHA* and *TPI1* gene. Both T_inter_ and T_term_ induced high expression of *PDPK1* and *SLC2A1*, whereas *FASN*, *GLS* and *TPP2* were similarly expressed by all three CD8^+^ T-cell subsets following PMA/ionomycin stimulation. In comparison to T_n_, we recorded higher expression of *HIF1A* and *SLC7A5* in T_inter_ and T_term_. Upon PMA/ionomycin stimulation, the highest levels of *SLC1A5*, *HK2* and *MYC* were expressed in T_n_, T_inter_ and T_term_, respectively. The ConA stimulation induced upregulation of ID2 expression in T_n_ and *SLC1A5* in T_inter_ only. Contrary, the PMA/ionomycin stimulation induced upregulation of *ID2* in all three CD8^+^ T-cell subsets (**Figure 3B**).

**Figure 4:**
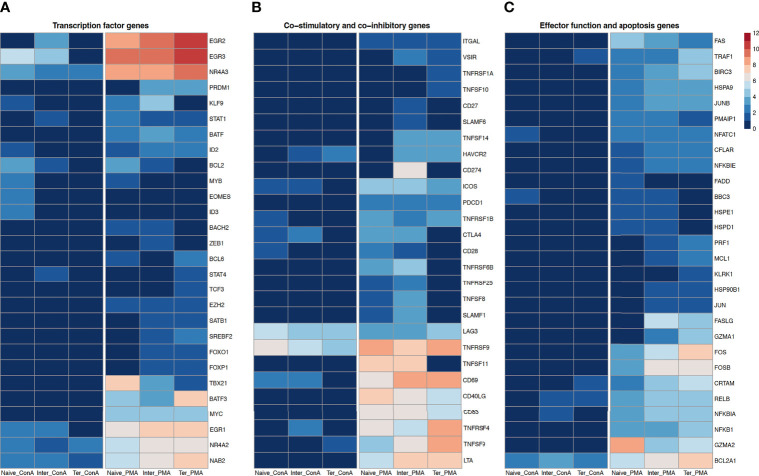
Transcription profiles of naïve, intermediate and terminally differentiated CD8^+^ T-cell subsets. Data derived from 18 ConA and 18 PMA/ionomycin-stimulated CD8^+^ T-cell subsets of six animals. As criteria to define DEGs, fold-change > |2| compared to medium control, maximum of the average RPKM’s > 2 and a false discovery rate corrected p-value < 0.01 (FDR) were used. **(A)** Expression of transcription factor genes as log2 fold-change between ConA- and PMA/ionomycin-stimulated CD8^+^ T-cell subsets and their unstimulated control. **(B)** Expression of co-stimulatory and co-inhibitory genes as log2 fold-change between ConA- and PMA/ionomycin-stimulated CD8^+^ T-cell subsets and their unstimulated control and **(C)** Expression of genes associated with effector functions and apoptosis as log2 fold-change between ConA- and PMA/ionomycin-stimulated CD8^+^ T-cell subsets and their unstimulated control.

Next, we examined the impact of stimulation with PMA/ionomycin and ConA on expression of transcription factor genes in CD8^+^ T-cell subsets (**Figure 4A**). Several genes encoding transcription factors associated with terminally differentiated effector cells, including *BATF*, *BATF3*, *EZH2*, *MYC* and *TBX21* were upregulated in all three CD8^+^ T-cell subsets upon PMA/ionomycin stimulation but not after ConA stimulation. While the highest expression of *BATF3*, *EZH2* and *MYC* was observed in the T_term_, highest upregulation of TBX21 which encodes the T-bet, the master regulator of cytotoxic T-cell development (45), was observed in the T_n_ compared to T_inter_ and T_term_. Interestingly, upregulation of *EOMES* and *ID3* was limited only to the ConA-stimulated T_n_. T_inter_ and T_term_ displayed upregulation of *FOXO1*, *FOXP1*, *PRDM1* (Blimp-1), *SATB1* and *SREBF2* following PMA/ionomycin stimulation. Recent studies in mice and human have shown that Blimp-1, encoded by *PRDM1*, enhances formation of SLECs, production of IL-10 and cytotoxic functions of CD8^+^ T cells (46). Along the PMA/ionomycin-stimulated differentiation subsets, we observed a gradual increase of expression of EGR family of zinc-finger transcription factors, including *EGR1*, *EGR2* and *EGR3*, which are upregulated upon TCR activation. Similar expression was recorded in case of *NAB2*, a coactivator and corepressor of T-cell function (47). Also, transcriptions of *NR4A2* and *NR4A3*, two members of the Nuclear receptor 4A (NR4A) family known for their important role during acute and chronic CD8^+^ T cell response (48), were highly expressed along the differentiation subsets. Moreover, stimulation with PMA/ionomycin induced the highest expression of both genes in T_term_, followed by T_inter_ and T_n_. In contrary, the highest expression of *NR4A2* and *NR4A3* in ConA-stimulated subsets was recorded in T_n_. Recently it has been reported that NR4A3 increases early expression of transcription factors involved in the SLEC differentiation and its absence favors differentiation of MPEC and central memory CD8^+^ T cells (49). Notably, T_n_ and T_inter_ but not T_term_ expressed high levels of *BCL2* upon PMA/ionomycin and ConA stimulation. These results are in accordance with the recent findings which show that naïve T cells highly express *BCL2* and are more dependent on it for survival than effector and memory T cells (50). The transcription factor *MYB* promotes formation of stem-like memory cell and restrains terminal effector differentiation by inducing expression of *BCL-2* and *TCF7* as well as inhibition of *ZEB2* (51). While T_n_ strongly expressed *MYB* following PMA/ionomycin and ConA stimulation, no upregulation was induced by T_inter_ or T_term_. Also, expression of *BACH2*, described as transcriptional repressor of terminal differentiation that restrains formation of short-lived effector cells (52, 53), was upregulated in T_n_ and T_inter_ but not T_term_ after PMA/ionomycin stimulation. Furthermore, the PMA/ionomycin stimulation induced the expression of *ZEB1* and *TCF3* in T_inter_ and T_term_, respectively. Studies in mice showed that *STAT1* and *STAT4* are important transcription factors for the clonal expansion and promotion of antigen-activated CD8^+^ T cells. Whereas *STAT1* effects type I IFN-dependent clonal expansion of CD8^+^ T cells, *STAT4* contributes to proliferation and effector maturation of CD8^+^ T cells triggered by IL-12-mediated signaling (54, 55). In fact, the PMA/ionomycin stimulation induced high expression of *STAT1* in all three CD8^+^ T-cell subsets, while the ConA stimulation induced the upregulation only in the T_inter_. In case of *STAT4* expression, we observed upregulation in the T_inter_ stimulated with ConA and T_term_ stimulated with PMA/ionomycin. Notably, while *BCL6* was highly expressed in T_n_ and T_term_ following PMA/ionomycin stimulation, expression of *ID2*, a transcriptional regulator upregulated by activated CD8^+^ T cells late in effector phase which can also influence their differentiation into memory cells (56), was upregulated in all three CD8^+^ T-cell subsets stimulated with PMA/ionomycin. Moreover, expression of *ID2* was also increased in the T_n_ following ConA stimulation. Transcription of *KLF9* in the T_n_ was increased with ConA and PMA/ionomycin stimulation as well as in PMA/ionomycin-stimulated T_inter_.

Looking at co-stimulatory genes, we found that expression of *CD27* (TNFRSF7), expressed mostly on naïve T cells and also required for T-cell memory in mice (35), was upregulated only in T_inter_ stimulated with PMA/ionomycin (**Figure 4B**). Also, another co-stimulatory gene *CD28*, which is absent from human effector CTLs (14), was upregulated in T_n_ and T_inter_ stimulated with PMA/ionomycin as well as in T_n_ stimulated with ConA. Furthermore, upon PMA/ionomycin stimulation all three CD8^+^ T-cell subsets expressed *ITGAL* (CD11a), a β2 integrin reported to be important for homing of T cells and generation of antigen-specific T cells (9). In all three CD8^+^ T-cell subsets PMA/ionomycin stimulation induced high expression of *CD40LG*, a member of the tumor necrosis factor superfamily transiently expressed on activated CD8^+^ T cells that promotes expansion and differentiation in a cell-extrinsic manner (57), and *CD83*, a member of the immunoglobulin superfamily. Expression of *CD69*, an early activation marker, was highly expressed in all three CD8^+^ T-cell subsets stimulated with PMA/ionomycin, and to a much smaller extent in T_n_ and T_inter_ upon ConA stimulation. These differences in transcripts of *CD69* concerning different stimulations can be explained with the fact that the *CD69* expression is upregulated already after 30 to 60 minutes after activation and declines promptly after 4-6 hours (58). Furthermore, transcription of the inducible T cell co-stimulator (*ICOS*), a member of the immunoglobulin family structurally close to CD28 and rapidly expressed on activated T cells (59), was highly increased in all three CD8^+^ T-cell subsets following PMA/ionomycin and to a lesser extent in T_n_ and T_inter_ after ConA stimulation. In addition, all three CTL subsets stimulated with PMA/ionomycin showed high increase of the lymphotoxin alpha (*LTA*), described to positively affect antigen-specific T-cell response during an acute LCMV infection through increase of IFN-γ production (60). Two members of the signaling lymphocytic activation molecule family (SLAMF), namely, *SLAMF1* and *SLAMF6*, were induced in T_n_ and T_inter_ after PMA/ionomycin stimulation. Highest expression of SLAMF1 and SLAMF6 has been reported on central memory and effector memory subsets of CD8^+^ T cells (61).

In case of co-inhibitory genes, known to inhibit T-cell activation, cytolytic function and cytokine production (62), we observed that expression of *PDCD1* (PD-1) was induced in all three CD8^+^ T-cell subsets stimulated with PMA/ionomycin, whereas its ligand *CD274* (PD-L1) was only expressed on T_inter_. Moreover, both T_n_ and T_inter_ showed upregulation of cytotoxic T lymphocyte antigen-4 (CTLA4) upon stimulations, with PMA/ionomycin stimulation inducing stronger expression. It has been shown that CTLA4 is closely related to CD28, binds to the same ligands (CD80 and CD86) and inhibits T cell response (63). Next, we found that expression of lymphocyte activation gene-3 (LAG3) was induced in all three CD8^+^ T-cell subsets after both stimulations. This is in accordance with previous research in mice suggesting that naïve CD8^+^ T cell show low expression of LAG3, but increase its expression in response to stimulation (64). Notably, expression of *HAVCR2*, which encodes T-cell immunoglobulin and mucin domain-3 (Tim-3) inhibitory molecule, was upregulated only in T_inter_ and T_term_. The tumor necrosis factor superfamily (TNFSF) and its corresponding receptor superfamily (TNFRSF) were differently expressed among porcine CD8^+^ T-cell subsets.

In case of TNFSFs, PMA/ionomycin stimulation induced expression of *TNFSF8* (CD30L) and *TNFSF11* (RANKL) in T_n_ and T_inter_, whereas transcript of *TNFSF14* (LIGHT) was upregulated in T_inter_ and T_term_. The highest expression of *TNFSF9* (4-1BBL) could be observed in T_term_, followed by T_inter_ and T_n_. In addition, the ConA stimulation induced its expression only in T_term_. Also, only T_term_ showed increased upregulation of *TNFSF10* (TRAIL) upon PMA/ionomycin stimulation. Regarding TNFRSFs, transcript of *TNFRSF1A* (TNFR1) was enriched in T_term_, while *TNFRSF1B* (TNFR2) was expressed in all three CD8^+^ T-cell subsets following PMA/ionomycin stimulation. Both T_n_ and T_inter_ expressed *TNFRSF6B* (DCR3) and *TNFRSF25* (DR3) after PMA/ionomycin stimulations. Notably, expression of *TNFRSF9* (4-1BB) was strongly induced in all CD8^+^ T-cell subsets upon both stimulations. Similarly, the PMA/ionomycin stimulation induced high expression of *TNFRSF18* (GITR) and *TNFRSF4* (OX40), an intermediate activation marker, in all CD8^+^ T-cell subsets, while stimulation with ConA induced the upregulation of these two genes in T_inter_ but only *TNFRSF18* in T_term_.

Genes associated with effector functions of CTLs were primarily highly expressed by T_term_, followed by T_inter_ and in just few cases by T_n_ following PMA/ionomycin stimulation (**Figure 4C**). Moreover, the ConA stimulation had almost no effect on upregulation of those genes in CD8^+^ T-cell subsets. Several genes linked to cytolytic activity, including *GZMA1*, *PRF1* (Perforin), *FASLG*, *JUN*, *MCL1* and *HSP90B1*, were upregulated only in T_inter_ and T_term_ following PMA/ionomycin stimulation. Also, the highest expression of genes belonging to Jun (*JUN*, *JUNB*) and Fos (*FOS*, *FOSB*) families was detected in the T_term_, followed by T_inter_ and T_n_. Similarly, expression of several other genes involved in effector function and apoptosis, including *BIRC3*, *CFLAR*, *CRTAM*, *HSPA9*, *NFATC1*, *NFKB1* and *NFKBIE* was induced among CD8^+^ T-cell subsets. Notably, only T_n_ and T_inter_ showed upregulation of *BBC3*, *HSPD1* and *HSPE1*. While killer cell lectin like receptor k1 (*KLRK1*) gene was upregulated in the T_term_, transcript of *FADD* was induced only in T_n_. Compared to T_inter_ and T_term_, we found higher expression of *GZMA2* and *PMAIP* in T_n_ stimulated with PMA/ionomycin. Interestingly, both ConA and PMA/ionomycin stimulation induced upregulation of *BCL2A1* in all CD8^+^ T-cell subsets, although with markedly stronger expression after PMA/ionomycin stimulation and in T_term_. What is more, the highest expression of *TRAF1* and *RELB* was observed in the T_term_, followed by T_inter_ and T_n_. In case of T_term_, expression of *TRAF1* and *RELB* was also induced by the ConA stimulation, whereas T_inter_ showed only the upregulation of *RELB* transcript. Expression of another IkB family gene linked to apoptosis (65), namely the *NFKBIA*, was highest in the T_inter_ stimulated with PMA/ionomycin.

### GO and Pathway Analysis of *Ex Vivo* CD8^+^ T-Cell Subsets

**Figure 5:**
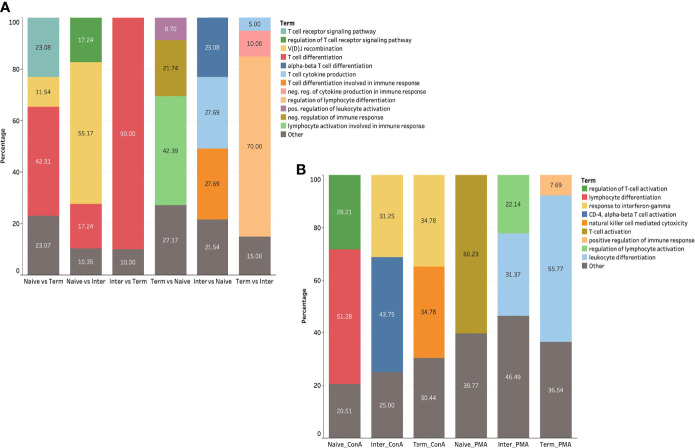
GO term analysis of *ex vivo* and *in vitro* stimulated CD8^+^ T-cell subsets **(A)** GO terms related to immune system of DEGs in *ex vivo* CD8^+^ T-cell subsets. **(B)** GO terms related to immune system of DEGs in CD8^+^ T-cell subsets upon ConA and PMA/ionomycin stimulation.

To extend the understanding of the immunological roles and functions of genes across different *ex vivo* CD8^+^ T-cell subsets, we performed GO term enrichment analysis. For GO terms related to immune system, most of the upregulated DEGs in the T_term_ were assigned to lymphocyte activation involved in immune response (42.39%) (**Figure 5A**). In contrast, upregulated DEGs in the T_n_ were mostly associated with T-cell differentiation (42.31%), T-cell receptor signaling pathway (23.08%) and V(D)J recombination (11.54%). The majority of upregulated DEGs in the T_inter_ compared to naïve subsets were related to T-cell differentiation involved in immune response (27.69%), T-cell cytokine production (27.69%) and alpha-beta T-cell differentiation (23.08%). On the other side, upregulated DEGs in the T_inter_ compared to T_term_ were mainly linked to the regulation of T-cell differentiation (90.0%). When compared to T_inter_, most of the upregulated DEGs in T_n_ were enriched for V(D)J recombination (55.17%), regulation of T-cell receptor signaling pathway (17.24%) and T-cell differentiation (17.24%), whereas within the T_term_ they were associated with regulation of lymphocyte differentiation (70.0%) (**Supplementary Table S2**, and **Figure S2**).

KEGG pathway analysis revealed that upregulated DEGs in the T_term_ compared to T_n_ were assigned to 272 pathways, including 21 pathways related to the immune system. Within immune-related pathways, the highest number of upregulated DEGs were enriched in chemokine signaling and T-cell receptor signaling pathways. Additionally, upregulated DEGs were linked to metabolic, MAPK signaling and cytokine-cytokine receptor interaction pathways. Compared to T_term_, DEGs within T_n_ were associated with 278 pathways, with 20 immune-related pathways, as well as metabolic and MAPK signaling pathways. When compared to T_inter_, DEGs of T_n_ and T_term_ were enriched in 192 and 167 pathways, respectively. Furthermore, DEGs of both T_n_ and T_term_ were enriched in 15 immune-related pathways. In comparison to T_n_ and T_term_, DEGs within T_inter_ were enriched in 268 and 235 pathways, respectively. All pathways including corresponding genes retrieved through KEGG pathway analysis were listed in **Supplementary Table S3**.

### GO and Pathway Analysis of *In Vitro* Stimulated CD8^+^ T-Cell Subsets

To further explore DEGs of *in vitro* stimulated CD8^+^ T-cell subsets, we conducted GO term enrichment analysis for immune system processes using previously mentioned bioinformatic software and plug-in package. The results demonstrated that the DEGs of PMA/ionomycin-stimulated T_n_ compared to medium control were mostly associated with the regulation of T-cell activation (60.23%) (**Figure 5B**). In contrast, the DEGs in PMA/ionomycin-stimulated T_term_ were differently linked to the leukocyte differentiation (55.77%). For the DEGs of PMA/ionomycin-stimulated T_inter_, we found enrichment in GO terms associated with the leukocyte differentiation (31.37%) and the regulation of lymphocyte activation (22.14%). Next, we investigated GO terms for immune processes in different CD8^+^ T-cell subsets stimulated with ConA, showing that the lymphocyte differentiation and the regulation of T-cell activation were more related with the T_n_ upon stimulation, while response to interferon-gamma term was typically associated with T_inter_ and T_term_ (**Supplementary Table S2** and **Supplementary Figure S2**).

We next performed KEGG pathway analysis of each CD8^+^ T-cell subset upon PMA/ionomycin and ConA stimulation as described above. Upregulated DEGs within PMA/ionomycin-stimulated T_n_ were assigned to 302 pathways in KEGG pathway database. Similar observations were made with T_inter_ (318 pathways) and T_term_ (305 pathways) CD8^+^ T-cell subsets. Although similar number of pathways related to immune system were observed among PMA/ionomycin-stimulated CD8^+^ T-cell subsets, a much higher number of DEGs was found from T_inter_ and T_term_ than T_n_. Furthermore, T_inter_ showed the highest number of DEGs enriched in T-cell receptor signaling pathway (36), followed by T_term_ (33) and T_n_ (25). Looking at the chemokine signaling pathway, T_inter_ and T_term_ showed same number of DEGs enriched in the pathway (26), whereas T_n_ had only 18 DEGs involved. Besides immune-related pathways, DEGs from all PMA/ionomycin-stimulated CD8^+^ T-cell subsets were highly enriched in metabolic, MAPK-signaling and cytokine-cytokine receptor interaction pathways. Interestingly, number of DEGs enriched in MAPK-signaling pathway was gradually increased along the CD8^+^ T-cell subsets. Based on upregulated DEGs upon ConA stimulation, we recorded 175 pathways in case of T_n_, 120 pathways for T_inter_ and only 43 pathways for T_term_. Also, upregulated DEGs within the T_n_ showed the highest number of immune-related pathways. In contrast to PMA/ionomycin-stimulated CD8^+^ T-cell subsets, ConA stimulation induced a limited number of genes associated with metabolic and MAPK-signaling pathways. Moreover, in all CD8^+^ T-cell subsets only few DEGs were enriched in T-cell receptor signaling, chemokine signaling and cytokine-cytokine receptor interaction pathways after ConA stimulation (**Supplementary Table S3**).

## Discussion

In the present study we assessed the transcriptome of three subsets within the CD8β^+^ T-cell population we hypothesize to represent distinct differentiation stages through RNA-Seq analysis. We aimed to identify differences in gene expression profiles between subsets as well as upon *in vitro* stimulation with ConA and PMA/ionomycin. Based on surface expression of CD11a and CD27, we defined differentiation stages of CD8β^+^ T-cells as follows: naïve (T_n_; CD8β^+^CD27^+^CD11a^low^), intermediate differentiated (T_inter_; CD8β^+^CD27^dim^CD11a^+^), and terminally differentiated cells (T_term_; CD8β^+^CD27^-^CD11a^high^). To the best of our knowledge, this is the first study which comprehensively describes the transcriptomes of porcine CD8^+^ T-cell subsets.

Differential gene expression analysis of *ex vivo* CD8^+^ T-cell subsets revealed significant differences between subsets regarding expression of genes associated with early and late stages of differentiation. By comparing T_n_ and T_term_ *ex vivo*, we found 575 and 709 DEGs upregulated, respectively. In particular, T_n_ highly expressed a set of genes encoding transcription factors, such as *LEF1*, *BACH2*, *TCF7* (TCF1), *SATB1*, *ZEB1* and *BCL2*, which maintain quiescence state (11, 12). In contrast, T_inter_ and T_term_ showed upregulation of transcription genes that drive terminally effector cell differentiation including *TBX21* (T-bet), *PRDM1* (Blimp-1), *ZEB2*, *ZNF683* (Hobit), *ID2* and *STAT4* (12, 30, 66). Moreover, we observed upregulation of genes related to effector function, cytokines and chemokines along the differentiation gradient. For example, expression of CX3CR1, receptor of Fractalkine/CX3C ligand 1 which expression correlates with the grade of effector CD8^+^ T differentiation (67), was highly induced on T_term_ and T_inter_. In previous studies in human and mice, Gerlach et al. identified three distinct effector subpopulations based on expression of CX3CR1, namely CX3CR1^-^, CX3CR1^int^ and CX3CR1^hi^. CX3CR1^hi^ CD8^+^ T effector cells were characterized as CD27^-^, CD127^-^, KLRG^+^, produced the smallest amount of IL2 and showed at least 50% higher expression of T-bet in comparison to CX3CR1^-^ and CX3CR1^int^ cells (68). Moreover, these values have been found to be typical for terminally differentiated T effector cells (7, 67) and are consistent with our findings of porcine T_term_.

In adult mice, naïve CD8^+^ T cell subpopulations are phenotypically characterized as CD11a^low^CD44^low^CD27^+^KLRG^-^CD62L^+^CD122^-^, while terminally differentiated effector cells (TTDE) are defined as CD11a^high^CD44^high^CD27^-^KLRG^+^CD62L^-^CD122^-^ (13). Besides expression of CD122 (*IL2RB*) in T_term_, this fits well with our findings on porcine T_n_ and T_term_. In addition, naïve CD8^+^ T cell subpopulations in mice show absence of *ITGA4* (CD49d), while it is highly expressed in more differentiated subpopulation such as CD8^+^ effector T cells, central and effector memory CD8^+^ T cells. Our values for *ITGA4* (CD49d) expression in T_inter_ and T_term_ correlate favorably with these previous reports and further support the idea of high *ITGA4* expression in more differentiated CD8^+^ T-cell subsets. In addition to the CD49d, mice antigen-experienced CTLs following LMCV infection express also CD11a (*ITGAL)* and Ki67 (*MKI67*) markers (10). Likewise, our data demonstrate high upregulation of *ITGAL* (CD11a) and *MKI67* (Ki67) in T_inter_ and T_term_ but not in the T_n_. A possible explanation for the differential expression of *MKI67* among porcine subsets is that T_inter_ and T_term_ are in the expansion phase of activated CD8^+^ T cells, which is accompanied by induced expression of the proliferation gene *MKI67*. Following expansion, antigen-experienced CD8^+^ T cells differentiate into SLEC (CD127^-^ KLRG1^+^) or MPEC (CD127^+^KLRG1^-^) exhibiting distinct functional profiles (7, 8). Whereas T_n_ showed high expression of *IL7R* (CD127) and low of *KLRG1*, we found the exact opposite expression of these genes in porcine T_inter_ and T_term_. Thus, between T_inter_ and T_term_, the expression of *KLRG1* was more than three times higher in T_term_, suggesting their more differentiated state. On the other hand, the expression of *IL7R* (CD127) was significantly higher in T_inter_ than T_term_. It can thus be reasonably assumed that T_inter_ and T_term_ may represent porcine MPEC and SLEC, respectively.

Our findings on high expression of *CD27*, *CCR7* and *CD28* in the T_n_ fit well with the four-dimensional model to address T-cell differentiation stages in human (13). In contrast, we found all three genes downregulated in the T_term_. Compared to T_term_, T_inter_ expressed *CD27* and *CD28*, but no *CCR7*, and based on this 4D model in humans they could represent in swine early-differentiated CD8^+^ T cells.

CTLs perform their main killing function through the release of granzymes and perforin as well as Fas ligand expression which induces apoptosis on the target cells (69). As expected, our analysis showed high expression of genes associated with cytolytic activity in the T_term_ and to lesser extent in T_inter_. Further analyses showed the highest upregulation of *GNLY*, *PRF1* (Perforin), *GZMB*, *FASL*, *INFG* and *TNF* in the T_term_ followed by the T_inter_. Moreover, our data confirmed an absence of these genes in *ex vivo* T_n_. Taken together, these results offer crucial evidences for different gene signatures of distinct CD8^+^ T-cell subsets.

As indicated by previous comparative studies on porcine, mice and human genome and transcriptome concerning immune system (70, 71), we also found higher numbers of orthologous genes shared between pig and human than shared by pig and mouse. In particular, from DEGs in T_n_ and T_term_ we found around 86% orthologs in human and 39% in mouse data sets of CD8^+^ T cells. These differences can be explained in part by the fact that pig and human share more orthologs, while mice show the highest number of unique immune response genes that are not present in human and pig (71–73). Therefore, our results provide additional support for the similarity between human and pig genome on immune level, highlighting the pig as an appropriate model for human immunology research.

In a parallel approach, we showed gene expression changes in CD8^+^ T-cell subsets upon stimulation with ConA and PMA/ionomycin. Generally, PMA/ionomycin stimulation induced much stronger upregulation of genes compared to stimulation with ConA. These additional results demonstrate two things. First, following PMA/ionomycin stimulation, CD8^+^ T-cell subsets acquired more similar gene expression profiles as indicated by high number of DEGs shared between CD8^+^ T-cell subsets. It is very likely that upon stimulation all three CD8^+^ T-cell subsets switch to an activated state and this is accompanied by functional changes in gene expression. Second evidence, although all three CD8^+^ T-cell subsets upregulated several genes associated with CD8^+^ T-cell activation and differentiation upon stimulation, the differences in gene expression profiles remained and they clustered into three distinct subsets again. Even though stimulated T_n_ expressed some genes associated with the T-cell activation and differentiation, including *TBX21*, *ID2*, *BATF* and *EZH2*, they still showed no expression of *GNLY*, *PRF1*, *GZMB* and *FASL* even after PMA/ionomycin stimulation. In some cases, they even induced upregulation of several genes linked to early stages of differentiation e.g. *BACH2* and *BCL6*, which negatively correlates with granzyme B expression in effector CD8^+^ T cells (74). Contrary to the findings on *in vitro* stimulated T_n_, we found even higher expression of late-stage differentiation genes in T_term_ and T_inter_ following *in vitro* stimulation. It may be assumed that T_n_ require more time to reach full cytotoxic potential, whereas T_inter_ and T_term_ promptly show cytotoxic activity and effectively produce cytokines upon *in vitro* stimulation.

GO term enrichment analysis of *ex vivo* CD8^+^ T-cell subsets revealed that most of DEGs were involved in immunological processes associated with T-cell differentiation. Once stimulated, T_inter_ and T_term_ were mostly enriched in same GO terms, whereas T_n_ were linked to other GO terms related to the immune system. Nevertheless, DEGs of T_n_ stimulated with ConA and PMA/ionomycin were enriched in differentiation and T-cell activation, respectively. This GO term enrichment analysis implies that T_inter_ and T_term_ share more comparable gene expression profile and functions compared to T_n_.

Furthermore, KEGG pathway analysis of DEGs in the *ex vivo* T_n_ and T_term_ were assigned to 278 and 272 pathways, respectively. Although DEGs of T_n_ and T_term_ were involved in similar number of immune-related pathways, we found higher number of DEGs of T_term_ represented in those pathways, including T-cell receptor and chemokine signaling pathways. In contrast, the lowest number of KEGG pathways obtained from DEGs between two subsets were found in case of T_inter_ and T_term_. In our view the results emphasize the differences in gene expression profiles among *ex vivo* CD8^+^ T-cell subsets, with biggest difference between T_n_ and T_term_ and smallest between T_inter_ and T_term_. As anticipated, PMA/ionomycin-stimulated CD8^+^ T-cell subsets were involved in much higher number of pathways than after ConA stimulation. Interestingly, for the T_term_, over seven times more KEGG pathways were obtained after PMA/ionomycin stimulation in comparison to ConA stimulation. In addition, a higher number of DEGs from T_inter_ and T_term_ were enriched in immune-related pathways than T_n_, which confirmed our initial findings on *ex vivo* CD8^+^ T-cell subsets. On the other hand, following ConA stimulation, the highest number of KEGG pathways was recorded in the T_n_, followed by T_inter_. Whereas CD8^+^ T-cell subsets showed high enrichment in T-cell receptor, chemokine signaling and cytokine-cytokine receptor interaction pathways upon PMA/ionomycin stimulation, the number of those pathways was substantially smaller once CD8^+^ T-cell subsets were stimulated with ConA. Thus, our findings show clearly that PMA/ionomycin stimulation of CD8^+^ T-cell subsets induces much stronger cytolytic T-cell response than ConA stimulation and that the response was earlier and stronger in more differentiated than naïve CD8^+^ T cells.

In the present study we investigated transcriptomes of *ex vivo* CD8^+^ T-cell subsets and after *in vitro* stimulation. We obtained comprehensive results showing that substantial gene expression differences exist among phenotypically defined porcine CD8^+^ T-cell subsets. Therefore, this work can serve as valuable reference for gene expression profiling of differentiation stages of porcine CD8^+^ T-cell subsets. The findings will support future *in vivo* gene expression studies in healthy as well as in infected or vaccinated animals in order to get a more complete picture of differentiation stages of porcine CD8^+^ T-cell subsets, especially after antigen-specific activation. We are aware of the limitation of this study since only gene expression was analyzed without validation of protein expression data. This is due to the lack of specific monoclonal antibodies. Nevertheless, the present findings identified specific targets and thus help to solve the problem of non-existing monoclonal antibodies against the respective differentiation antigens.

## Data Availability Statement

The datasets presented in this study can be found in online repositories. The names of the repository/repositories and accession number(s) can be found below: https://www.ncbi.nlm.nih.gov/, PRJNA761916.

## Ethics Statement

Ethical review and approval was not required for the animal study because porcine blood was collected from abattoir in accordance with Austrian Animal Welfare Slaughter Regulation.

## Author Contributions

EL, MS, KM, and AS designed the project. EL performed lymphocyte isolation, *in vitro stimulation*, magnetic-activated cell sorting and RNA isolation. KM organized fluorescence-activated cell sorting. MV and SO prepared library and sequenced the samples. EL performed in-depth bioinformatic analysis. EL and AS analyzed the experiments and wrote the manuscript. CP assisted with interpretation of the data. All authors contributed to the article and approved the submitted version.

## Funding

This work was financially supported by intramural funds of the University of Veterinary Medicine Vienna.

## Conflict of Interest

MV and SO are employed by Istituto di Ricerche Biomediche “A. Marxer” RBM S.p.A. MS is employed by Merck Healthcare KGaA.

The remaining authors declare that the research was conducted in the absence of any commercial or financial relationships that could be construed as a potential conflict of interest.

## Publisher’s Note

All claims expressed in this article are solely those of the authors and do not necessarily represent those of their affiliated organizations, or those of the publisher, the editors and the reviewers. Any product that may be evaluated in this article, or claim that may be made by its manufacturer, is not guaranteed or endorsed by the publisher.
