# Supplementary material for: Transcriptome Profiling of Porcine Naïve, Intermediate and Terminally Differentiated CD8+ T Cells
Source: Front Immunol. 2022 Feb 21;13:849922. doi: 10.3389/fimmu.2022.849922 (PMC8900158; doi:10.3389/fimmu.2022.849922)
Supplement: Supplementary file 1 [file DataSheet_1.docx]

Supplementary Material

# Supplementary Figures

**
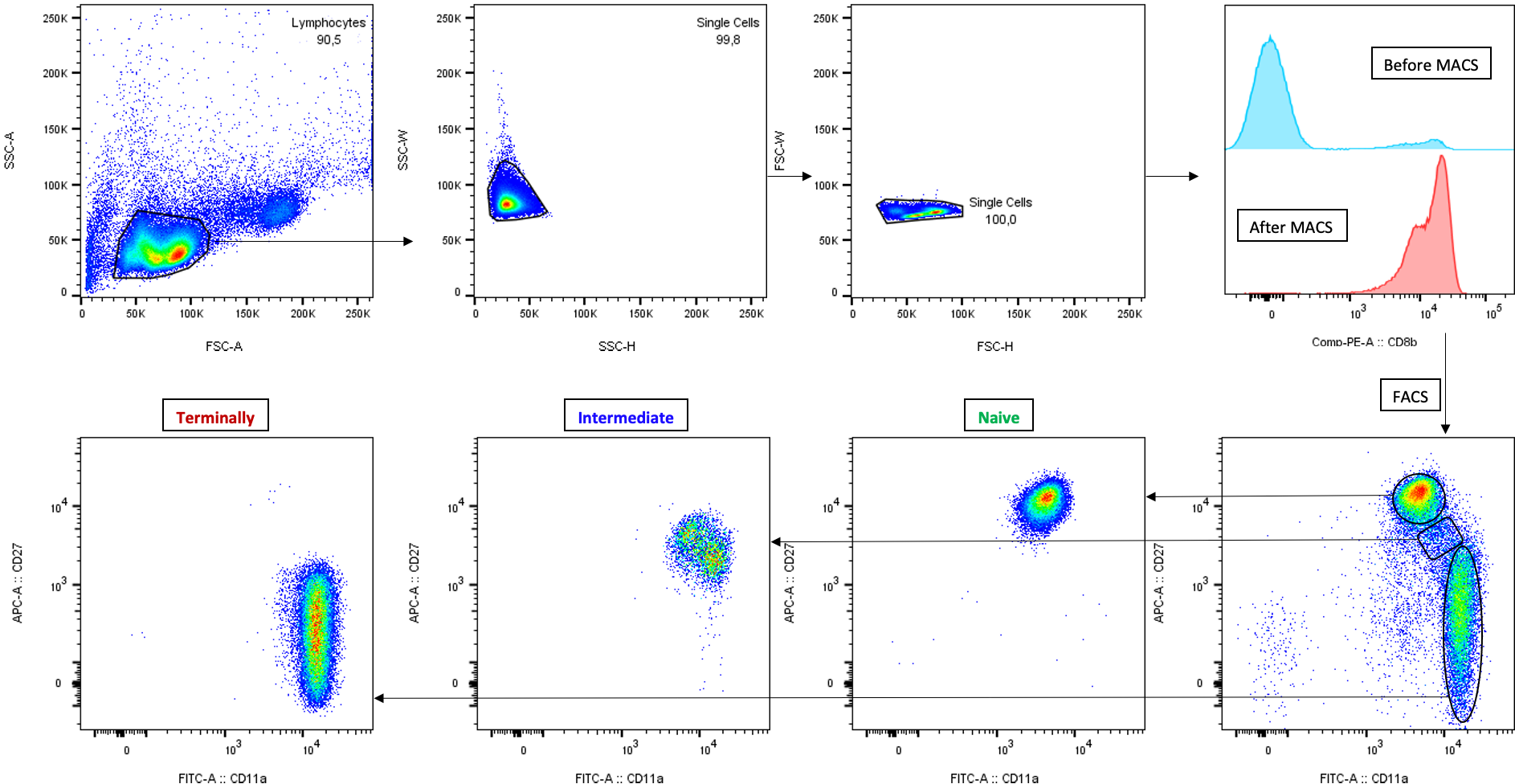
**

**Supplementary Figure 1.** **Consecutive gating strategy for lymphocytes and CTL subsets.** For each flow cytometry staining panel the following gating hierarchy was applied: lymphocytes were selected according to their light scatter properties (SSC-A vs. FSC-A). Thereafter, a two-step doublet discrimination (SSC-W vs. SSC-H and FSC-W vs. FSC-H) was applied, which was followed by the magnetic-activated cell sorting, based on a staining with the in-house produced primary monoclonal anti-CD8β antibody (clone PPT23, IgG1). Hereafter, CD8β^+^ cells were FACS sorted based on surface expression of CD27 and CD11a. Representative pseudocolor plots are shown (right to left): Naïve (CD8β^+^CD27^+^CD11a^low^); Intermediate differentiated cells (CD8β^+^CD27^dim^CD11a^+^); Terminally differentiated cells (CD8β^+^CD27^-^CD11a^high^).
