# Supplementary material for: Transcriptome Profiling of Porcine Naïve, Intermediate and Terminally Differentiated CD8+ T Cells
Source: Front Immunol. 2022 Feb 21;13:849922. doi: 10.3389/fimmu.2022.849922 (PMC8900158; doi:10.3389/fimmu.2022.849922)

# Overview of immune processes of DEGs in Naïve vs. Terminally CTLs

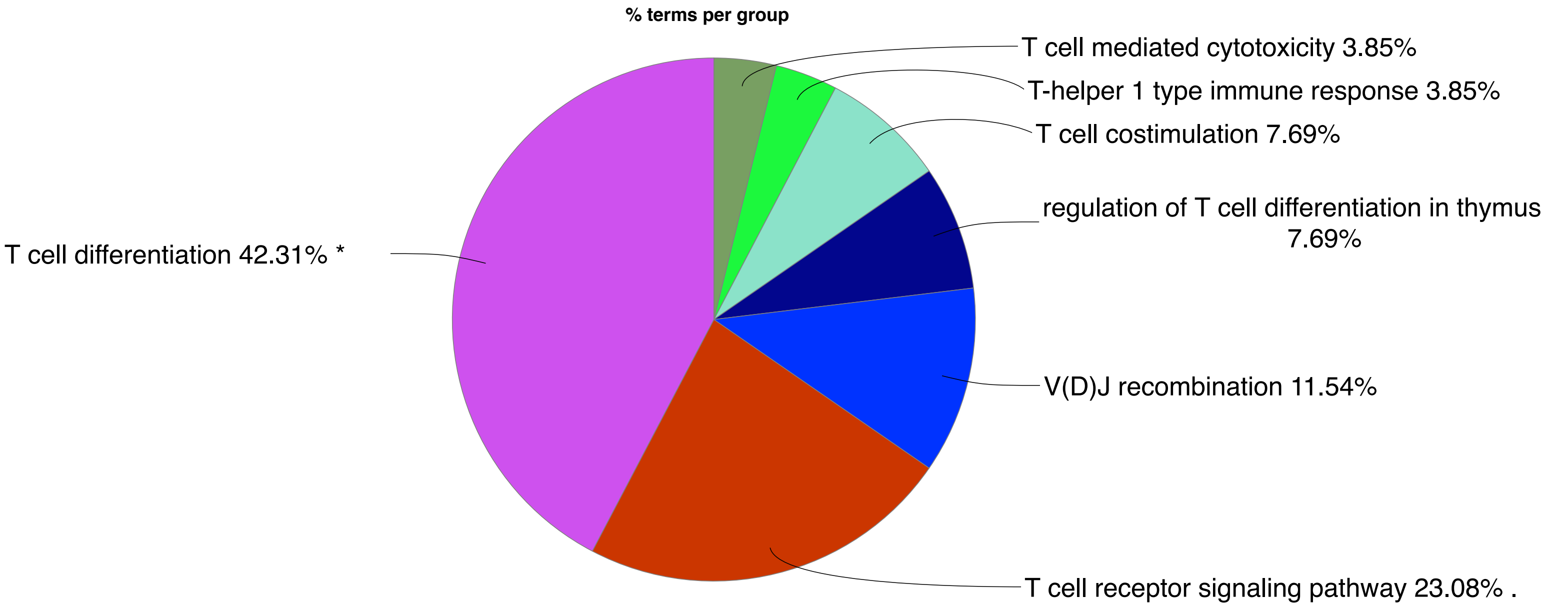

# Specific GO terms of immune processes of DEGs in Naïve vs. Terminally CTLs

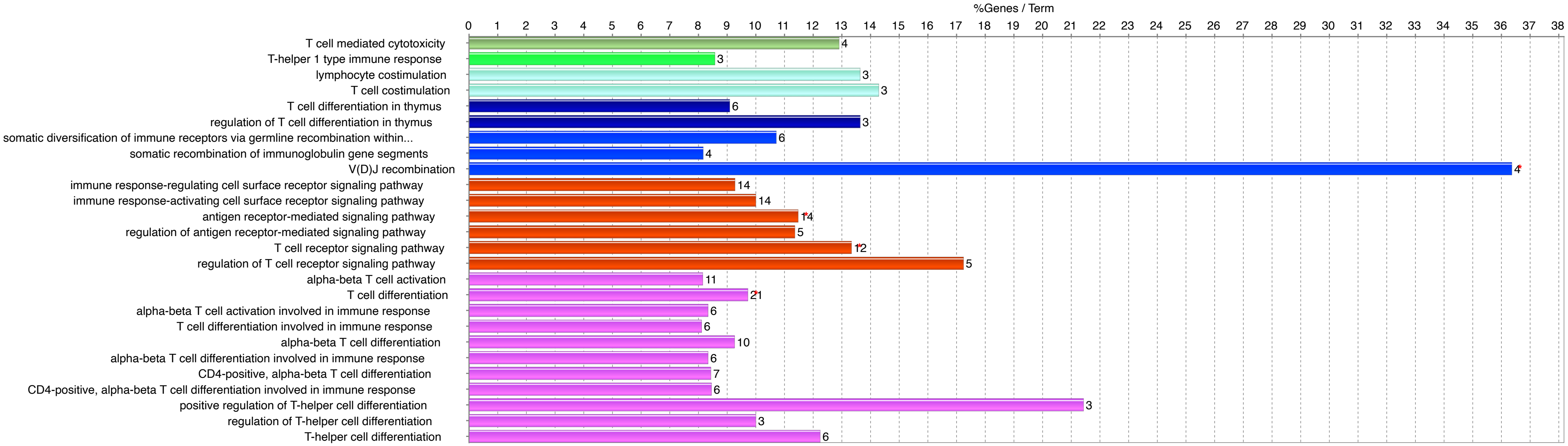

**Overview of immune processes of DEGs in Naïve vs. Intermediate CTLs**

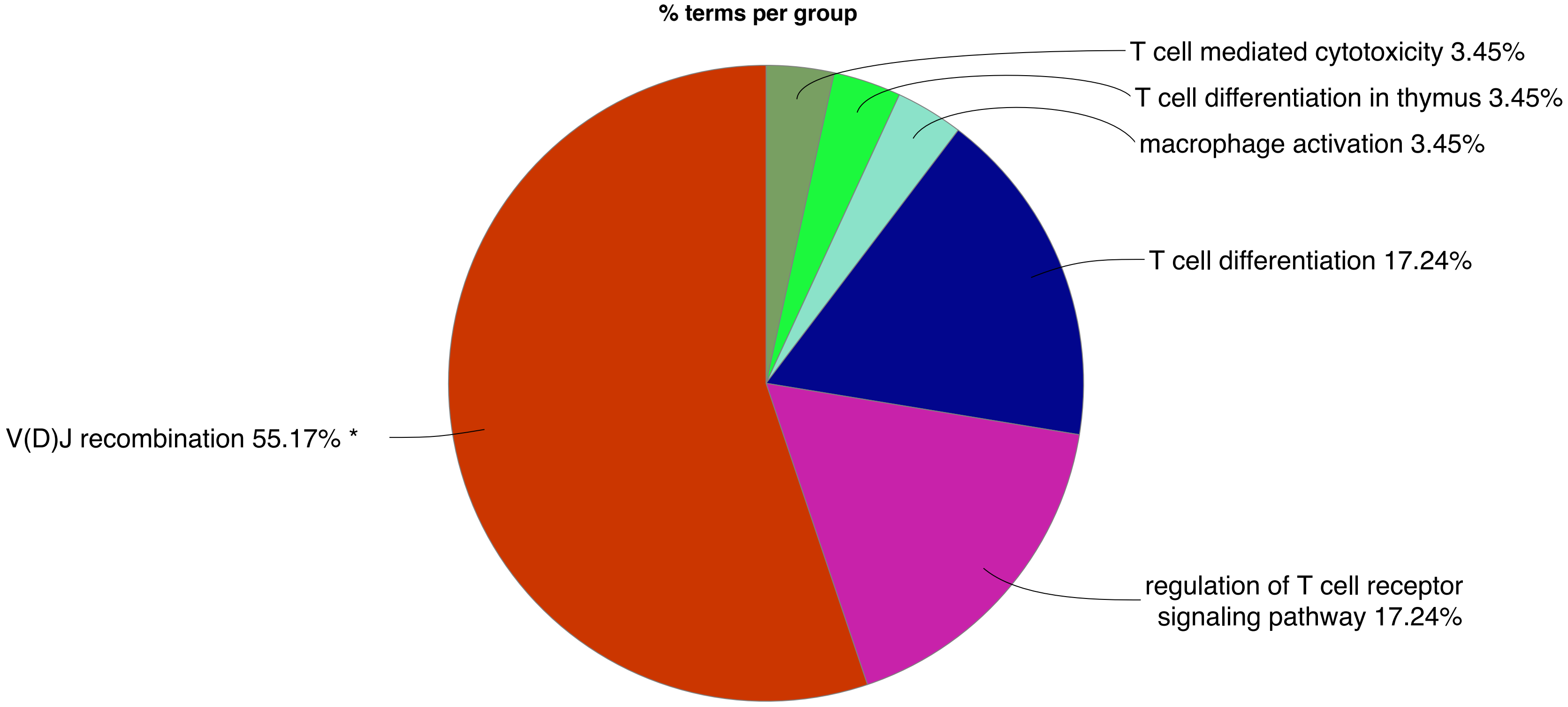

# Specific GO terms of immune processes of DEGs in Naïve vs. Intermediate CTLs

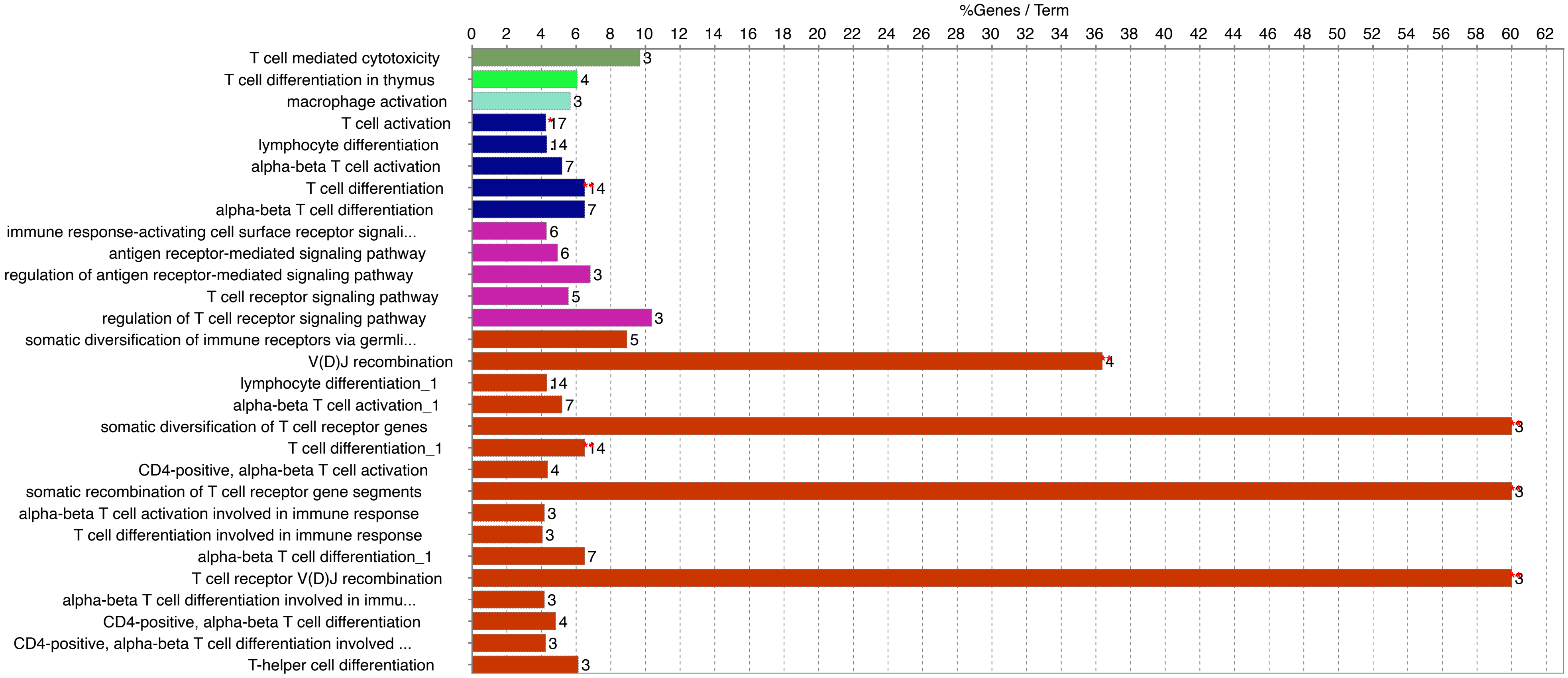

## Overview of immune processes of DEGs in Intermediate vs. Terminally CTLs

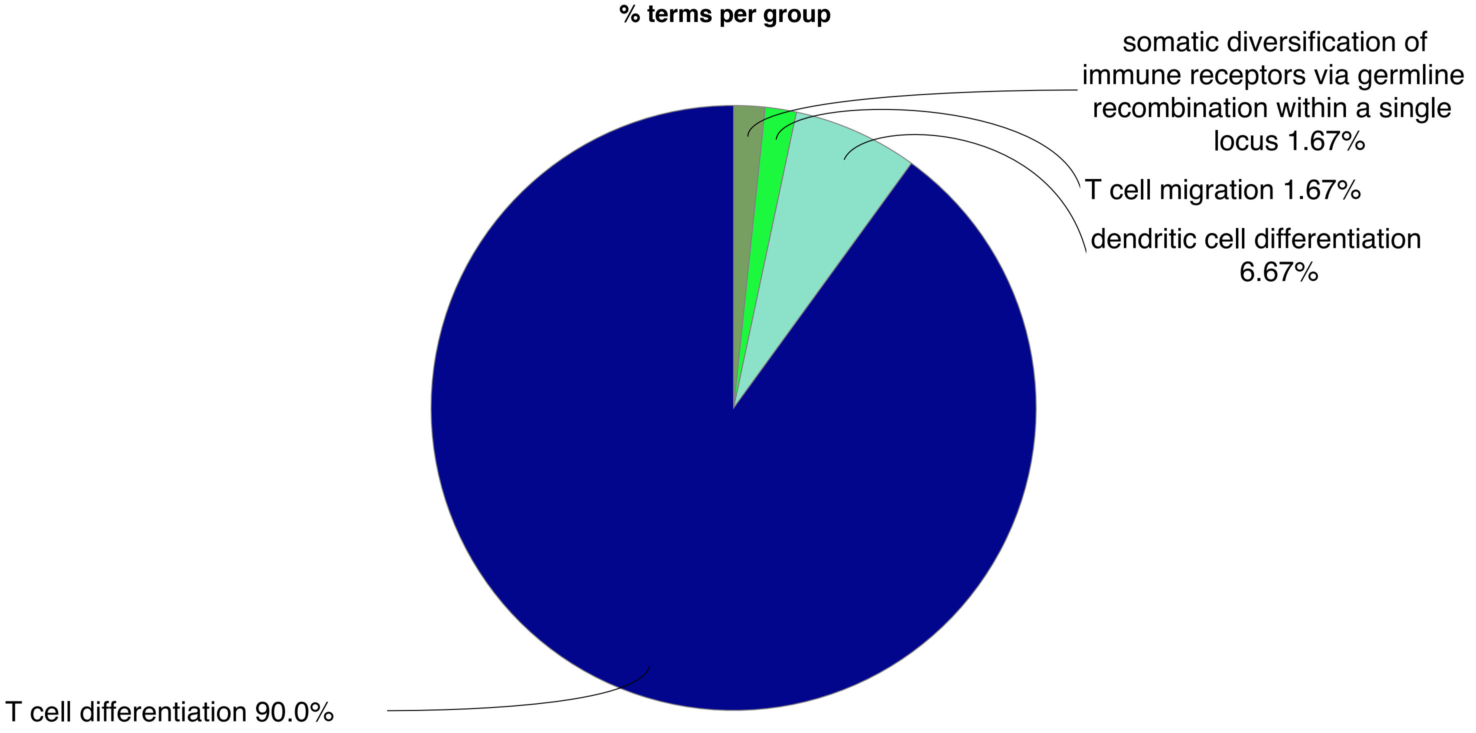

# Specific GO terms of immune processes of DEGs in Intermediate vs. Terminally CTLs

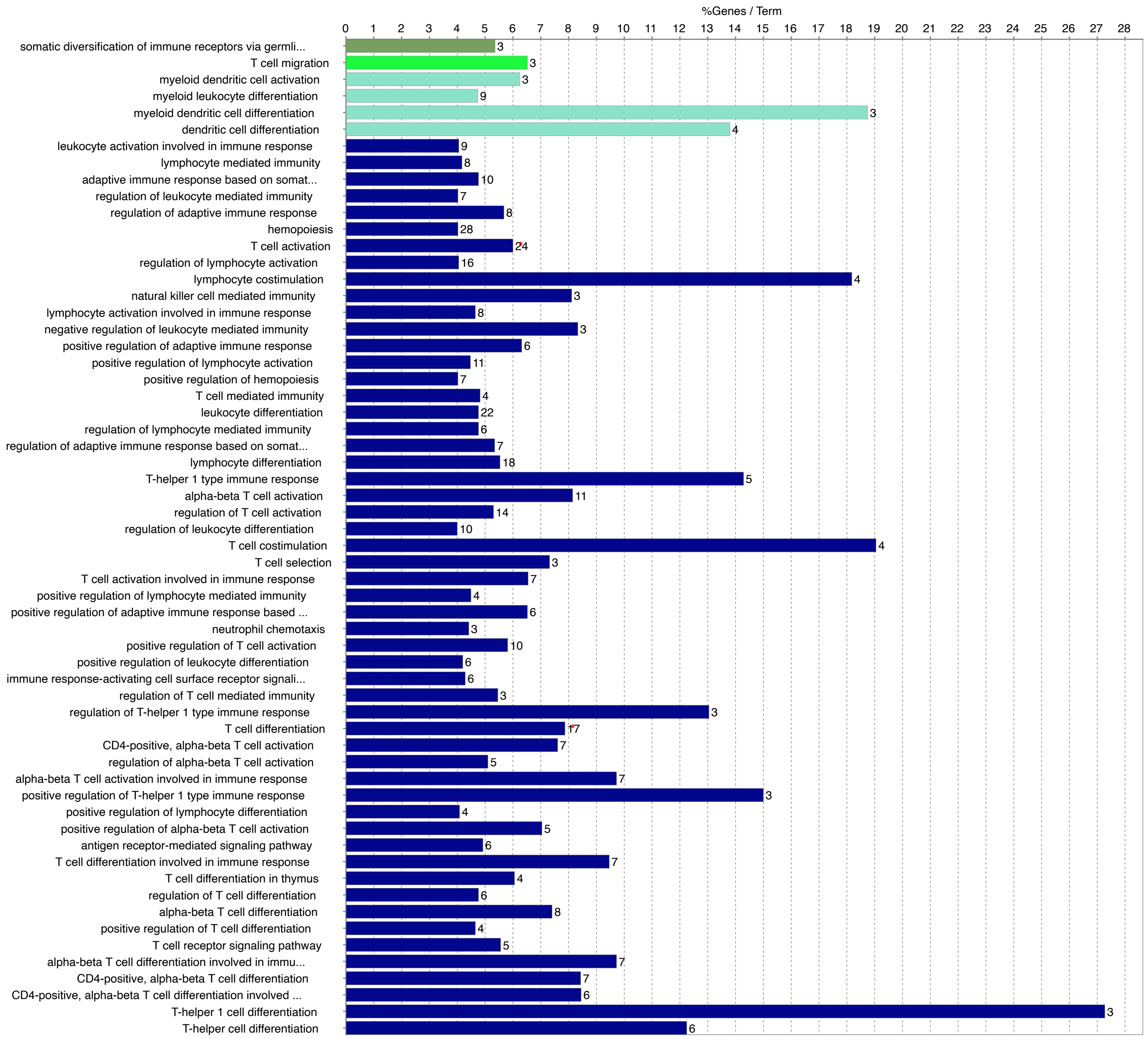

## Overview of immune processes of DEGs in Terminally vs. Naive CTLs

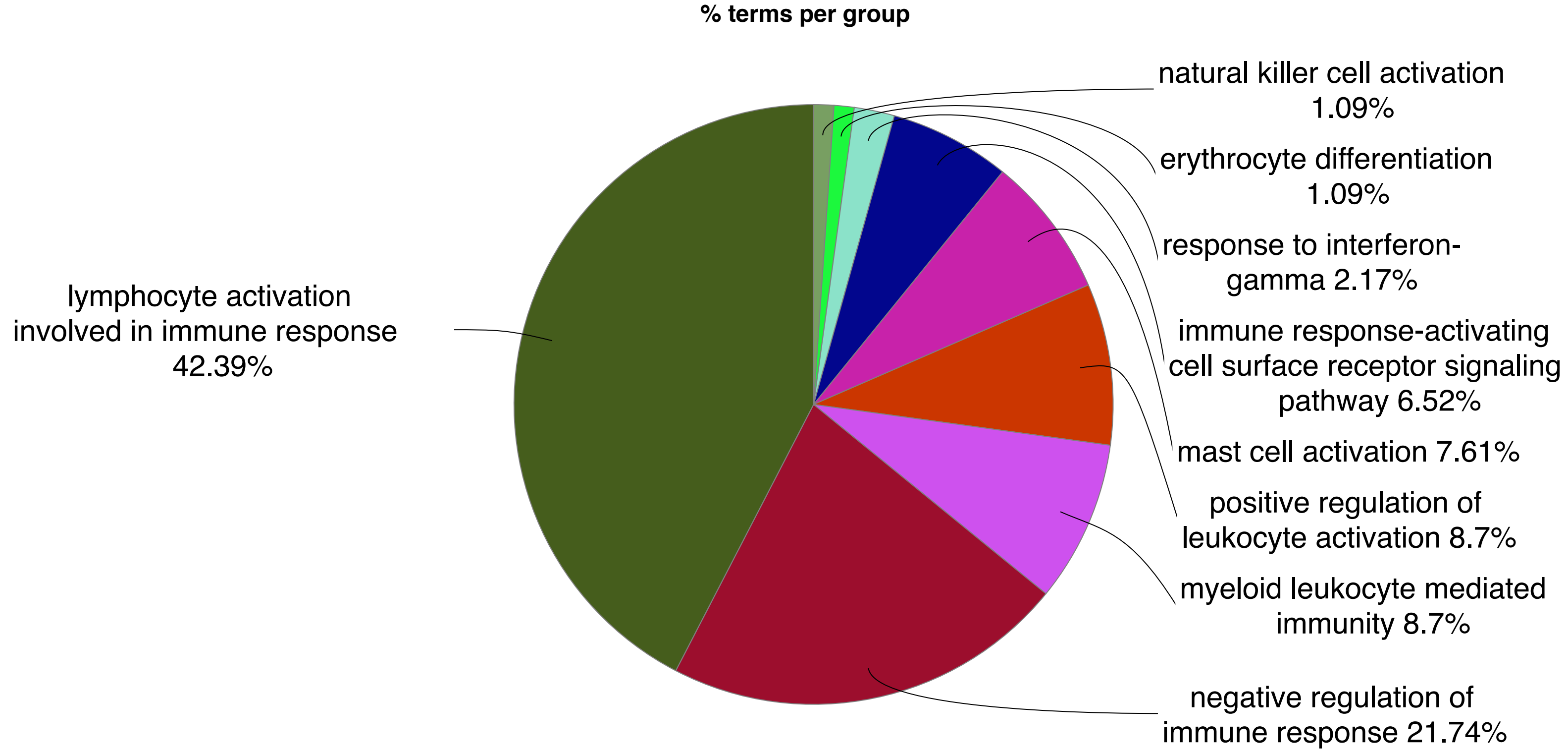

# Specific GO terms of immune processes of DEGs in Terminally vs. Naive CTLs

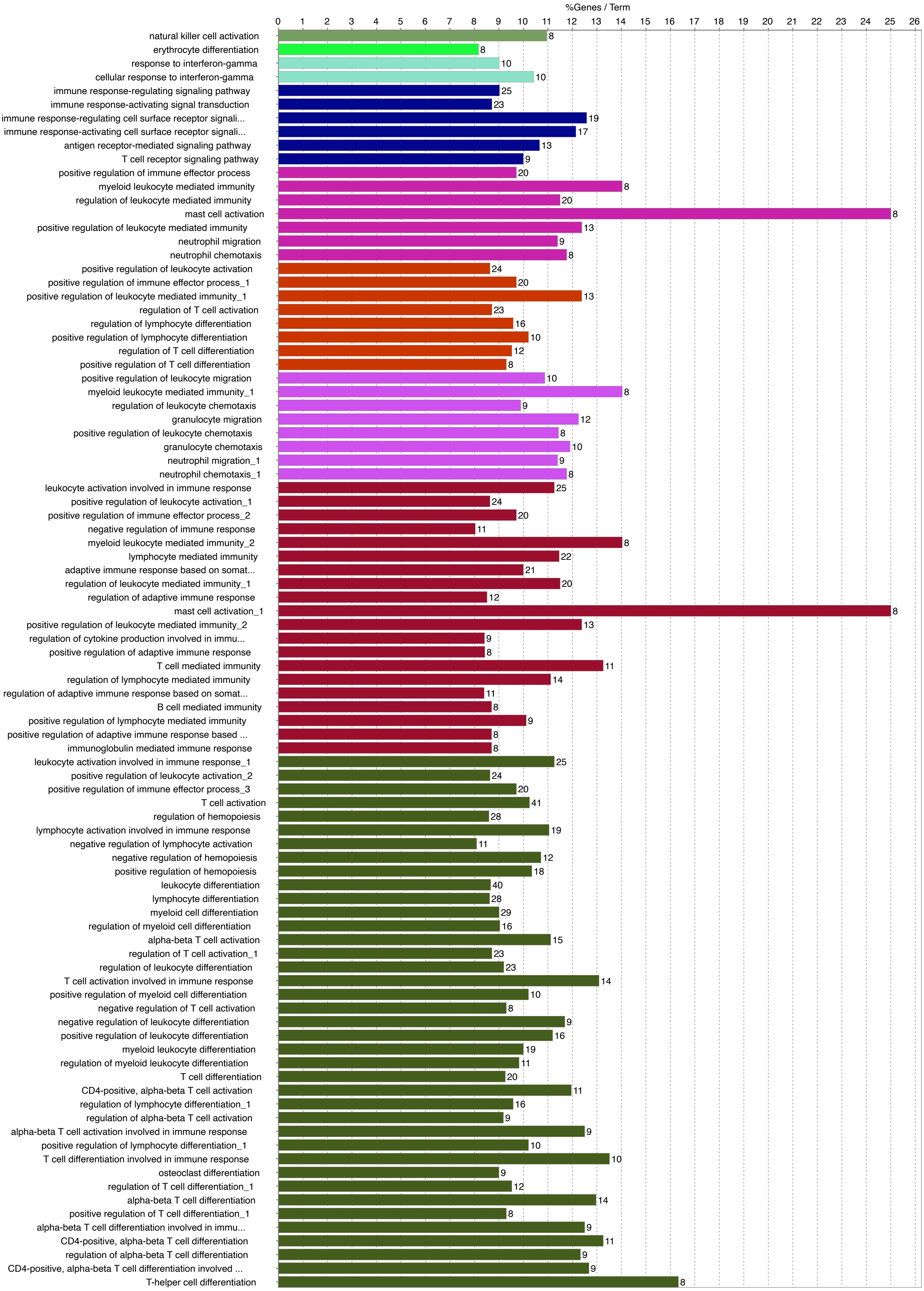

## Overview of immune processes of DEGs in Intermediate vs. Naive CTLs

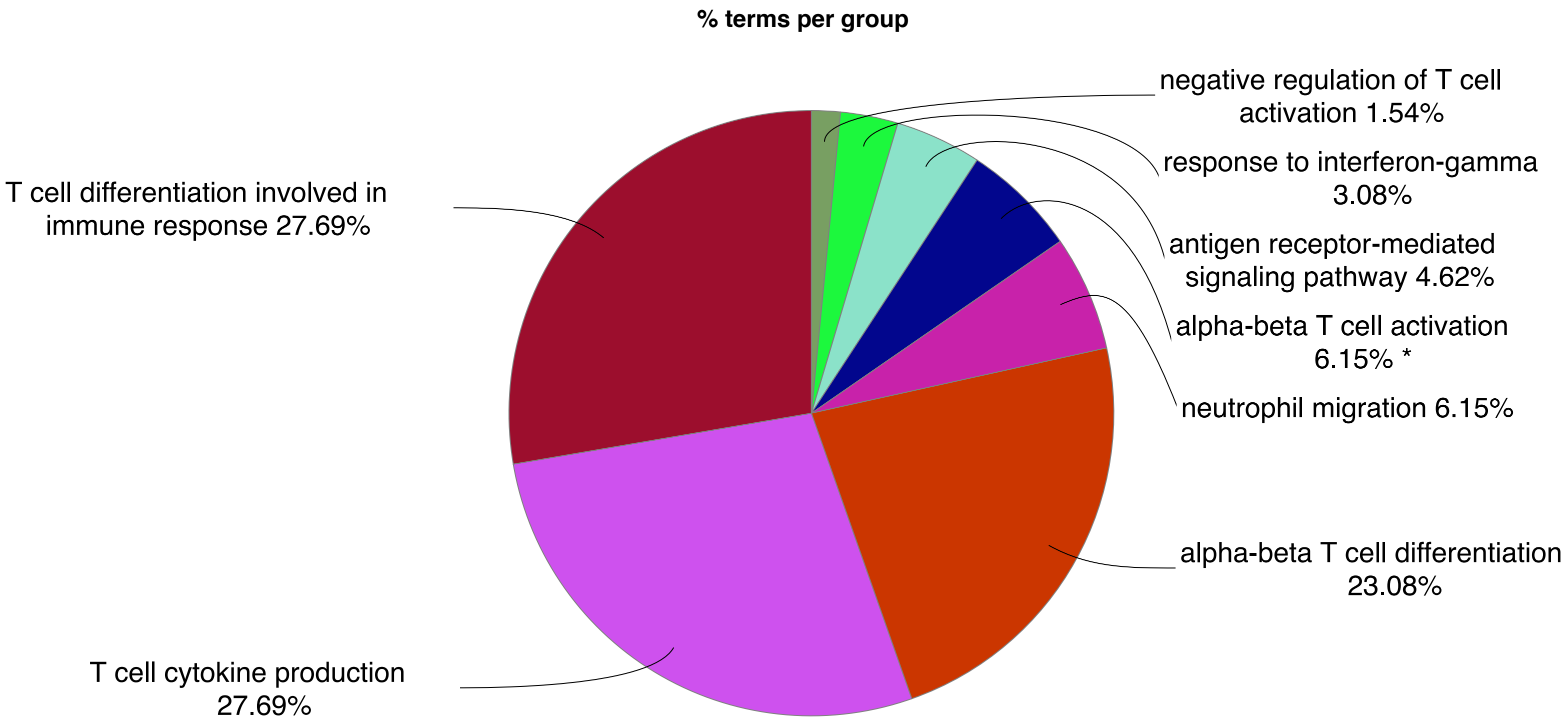

# Specific GO terms of immune processes of DEGs in Intermediate vs. Naive CTLs

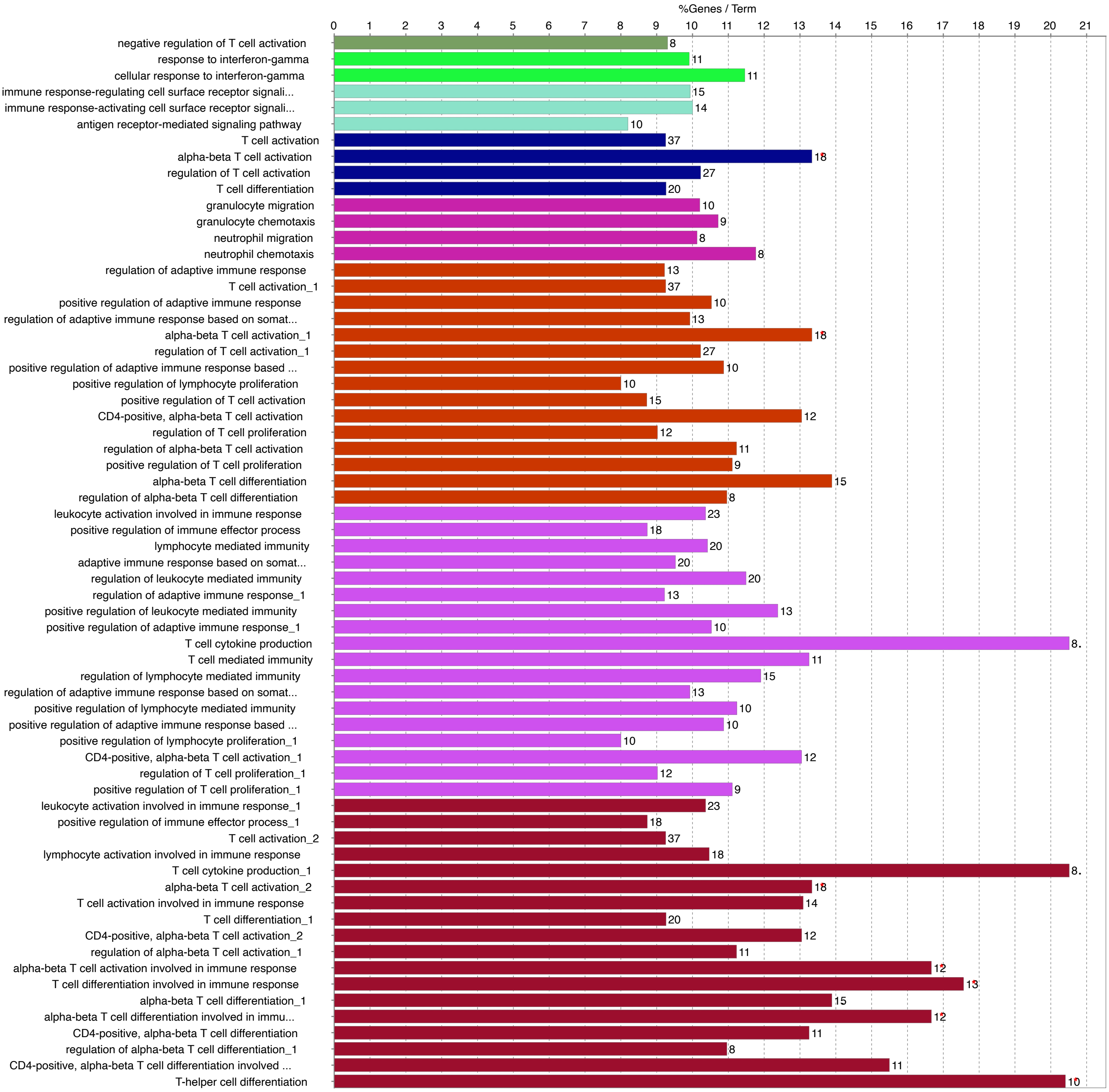

# Overview of immune processes of DEGs in Terminally vs. Intermediate CTLs

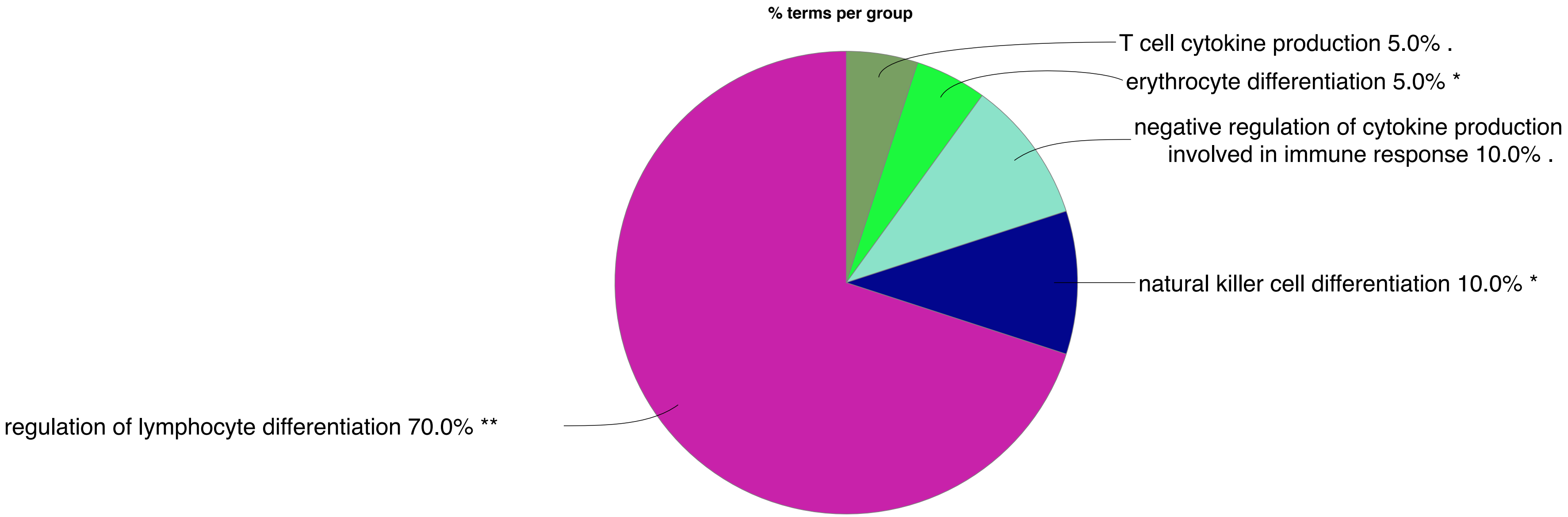

# Specific GO terms of immune processes of DEGs in Terminally vs. Intermediate CTLs

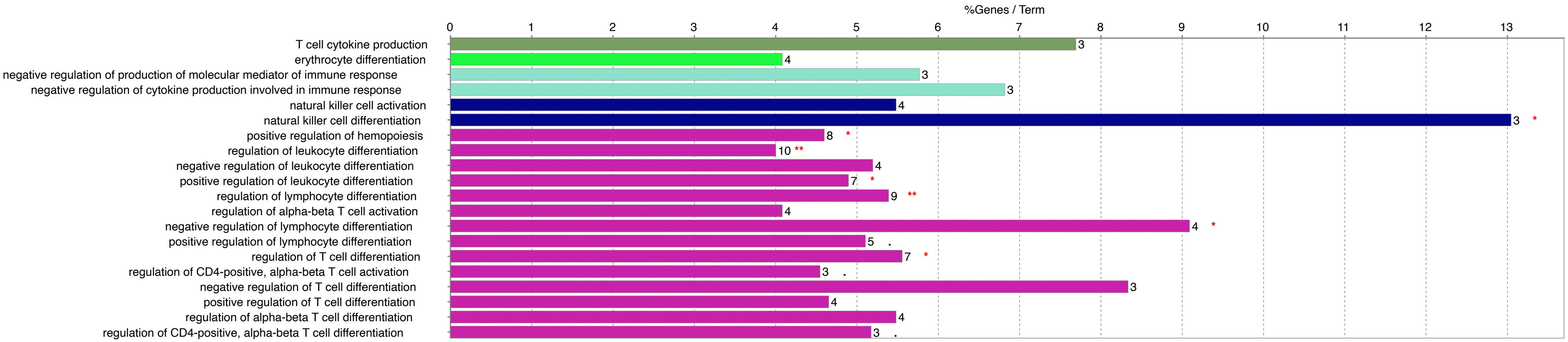

# Overview of immune processes of DEGs in Naïve_ConA

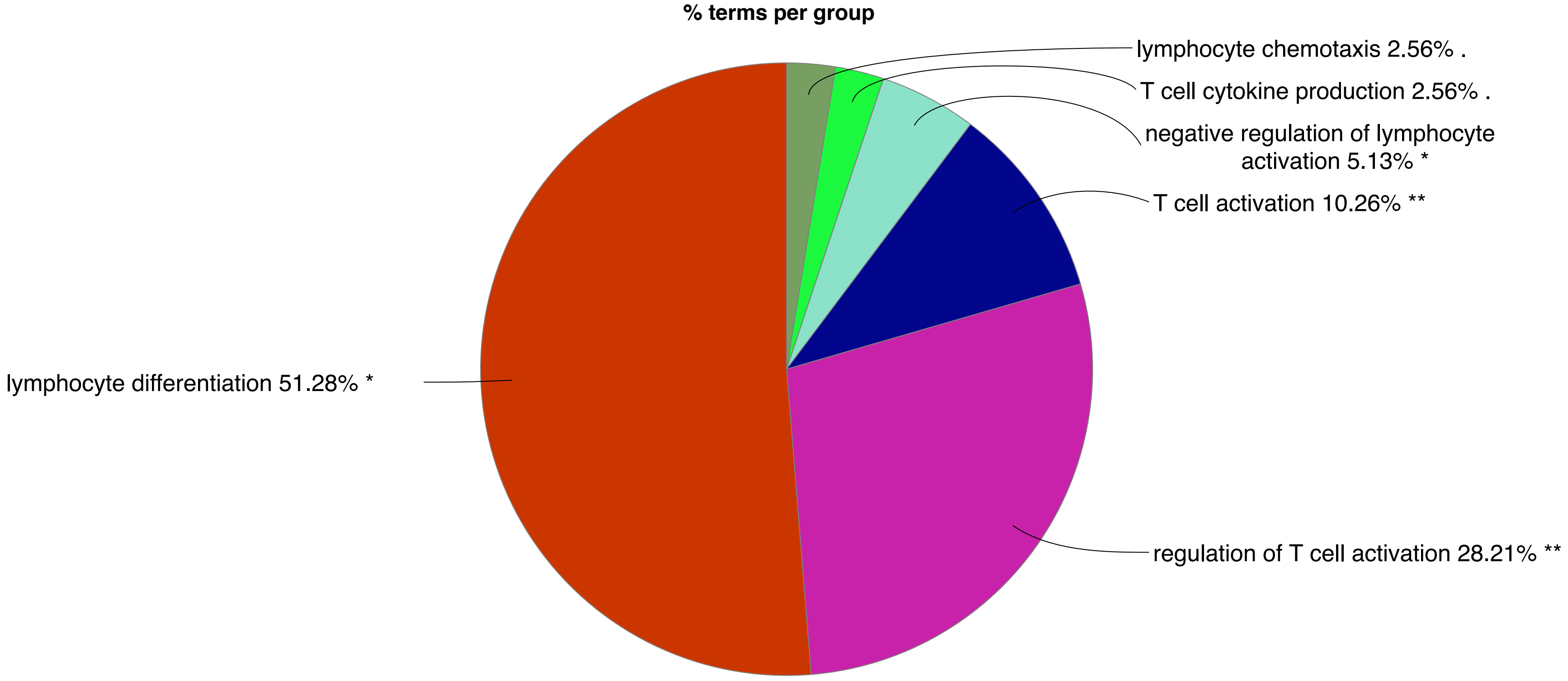

## Specific GO terms of immune processes of DEGs in Naïve_ConA

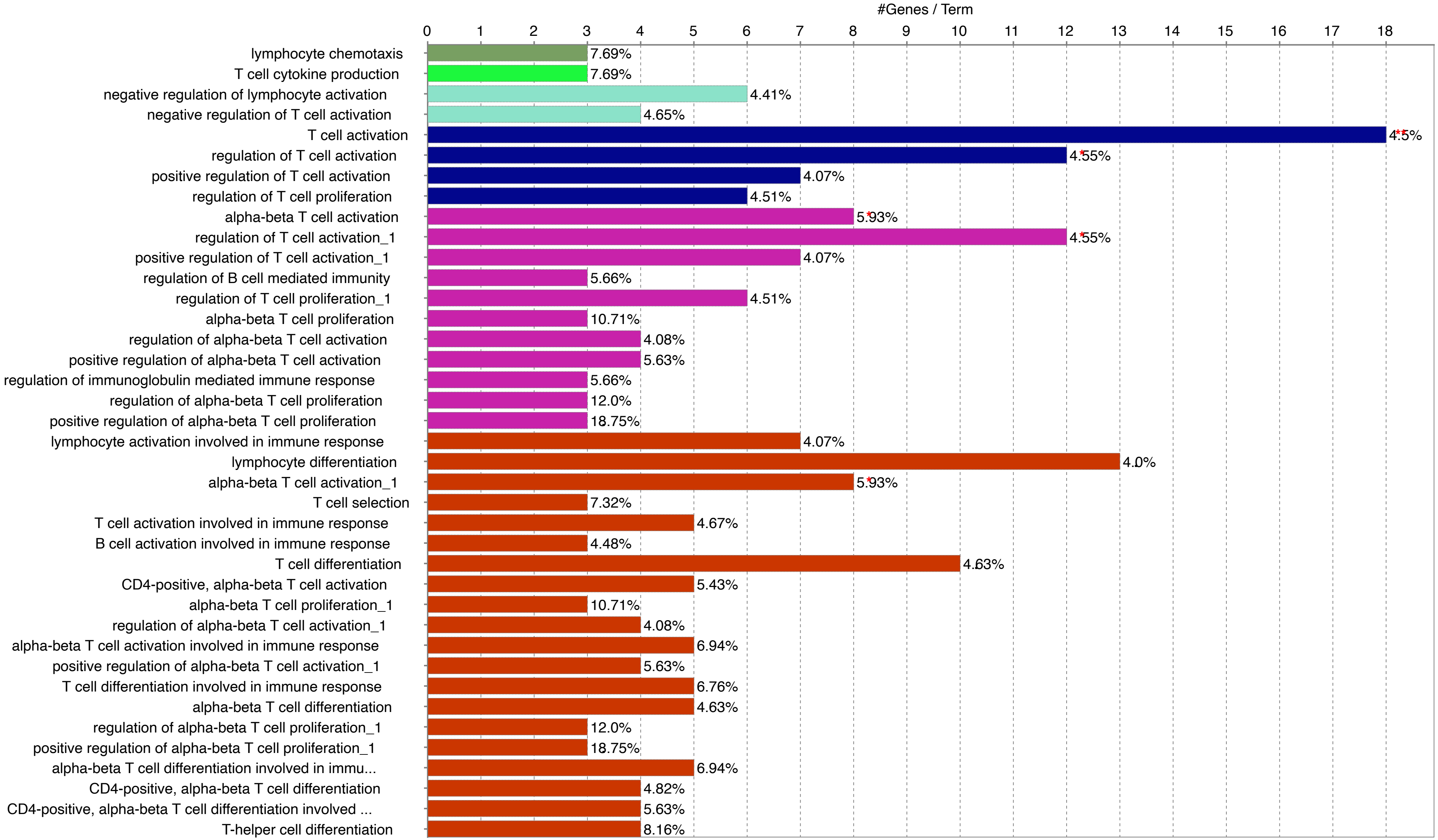

# Overview of immune processes of DEGs in Intermediate_ConA

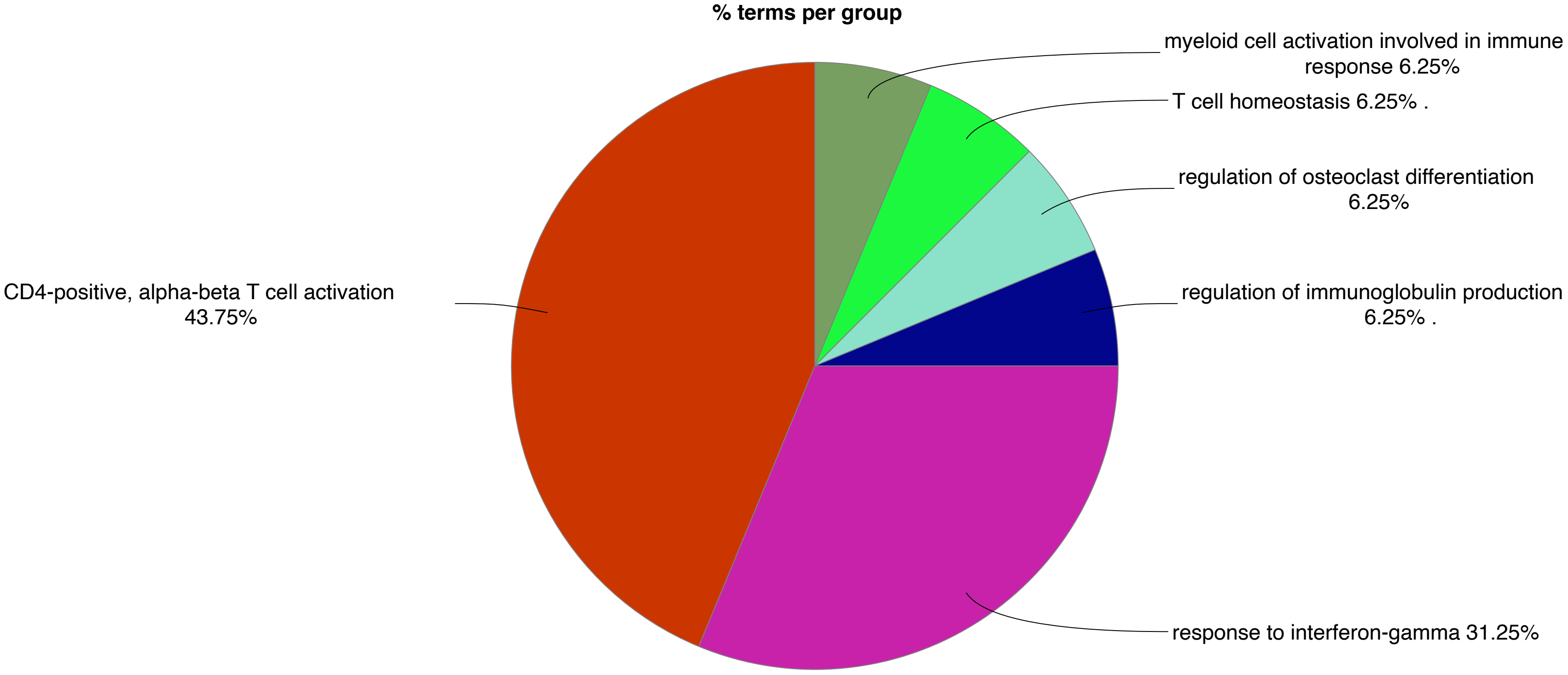

# Specific GO terms of immune processes of DEGs in Intermediate_ConA

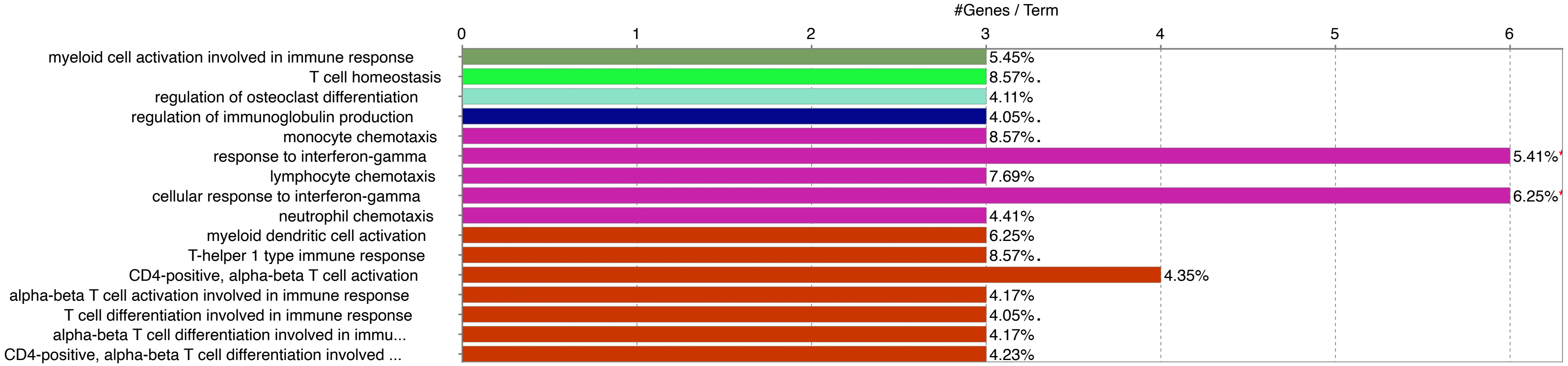

**Overview of immune processes of DEGs in Terminally_ConA**

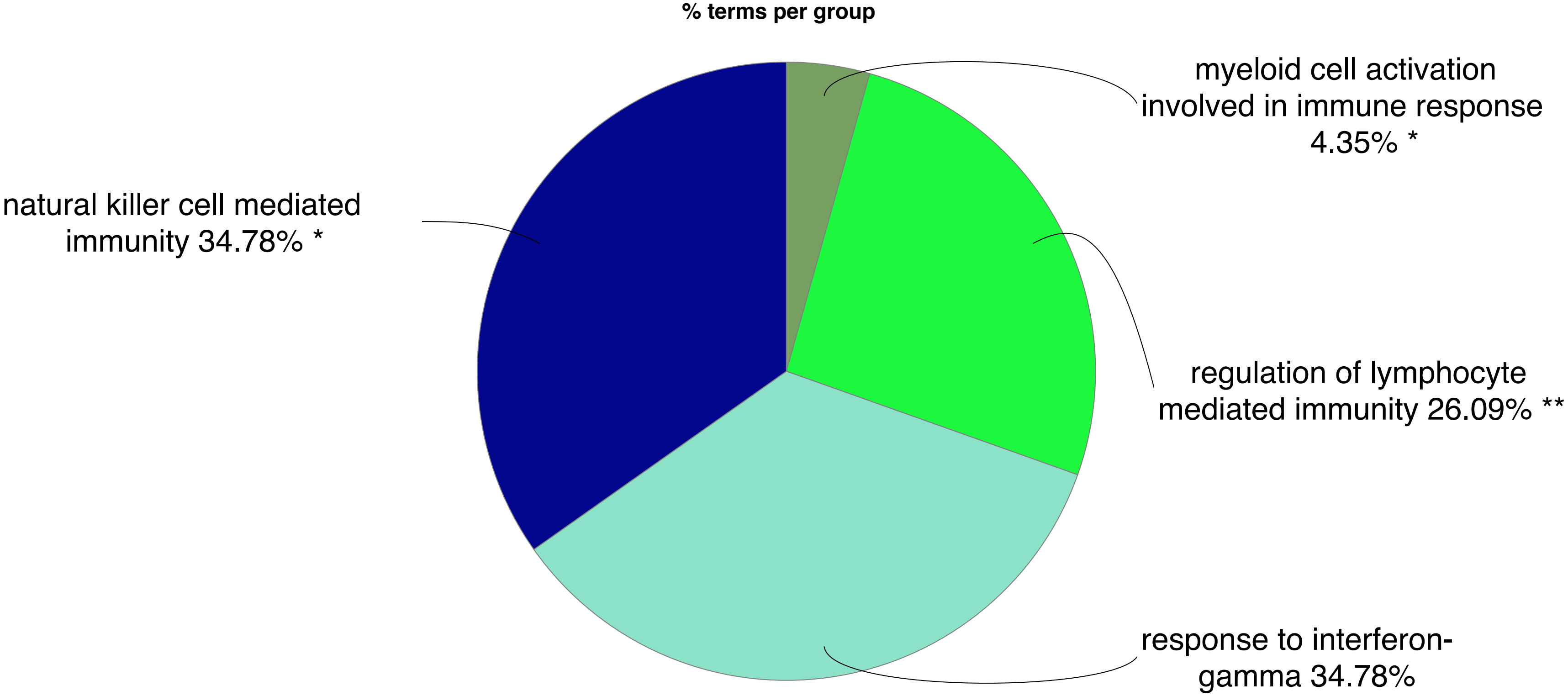

# Specific GO terms of immune processes of DEGs in Terminally_ConA

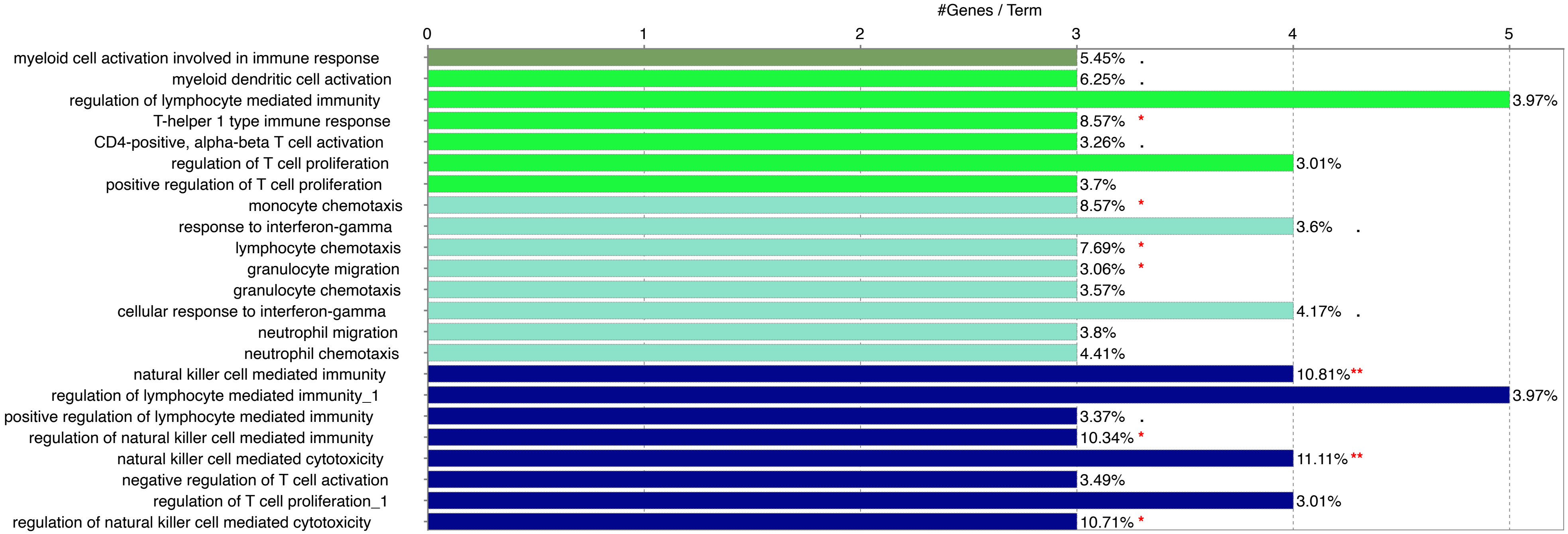

# Overview of immune processes of DEGs in Naïve_PMA/Ionomycin

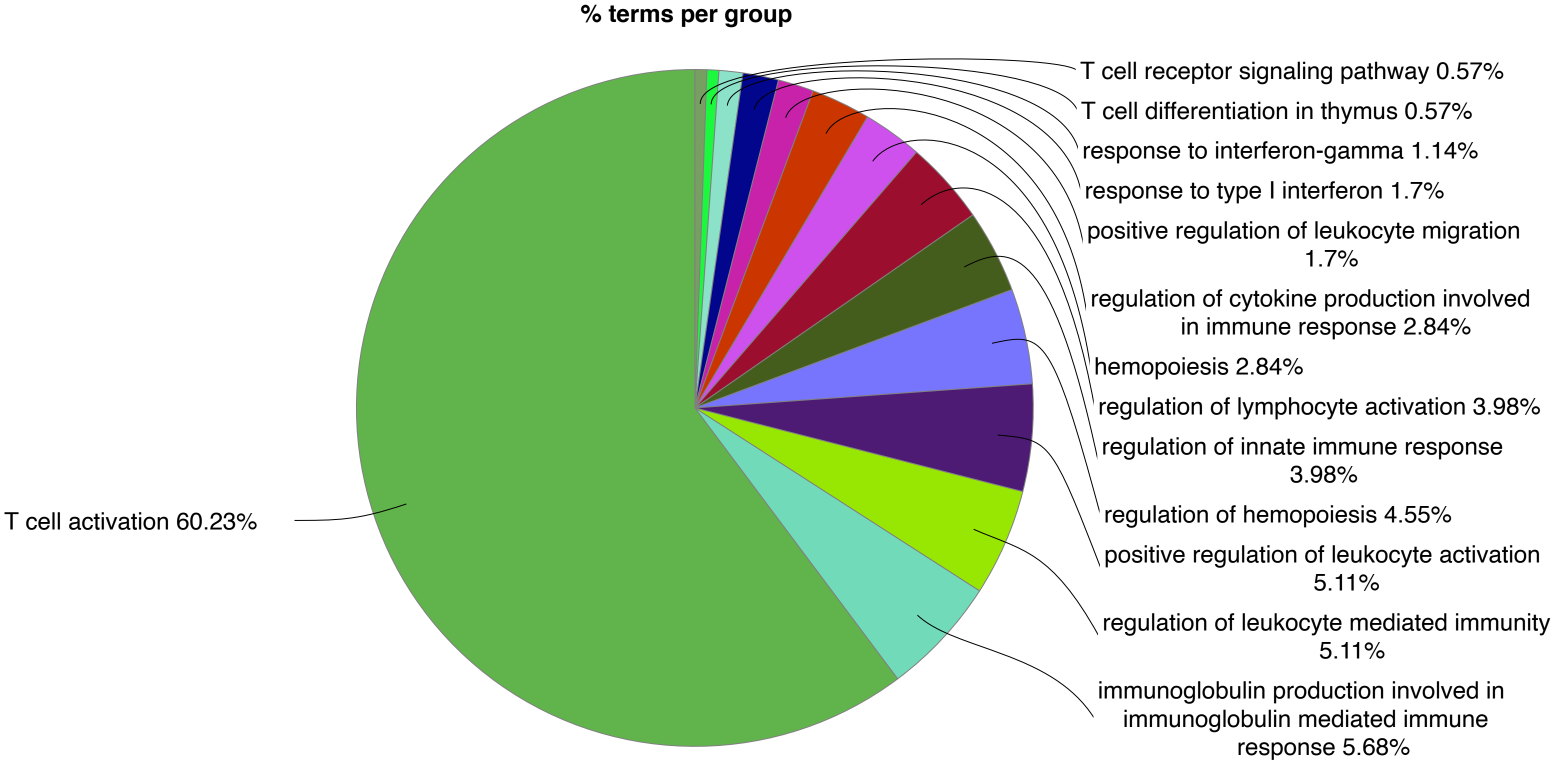

# Specific GO terms of immune processes of DEGs in Naïve_PMA/Ionomycin

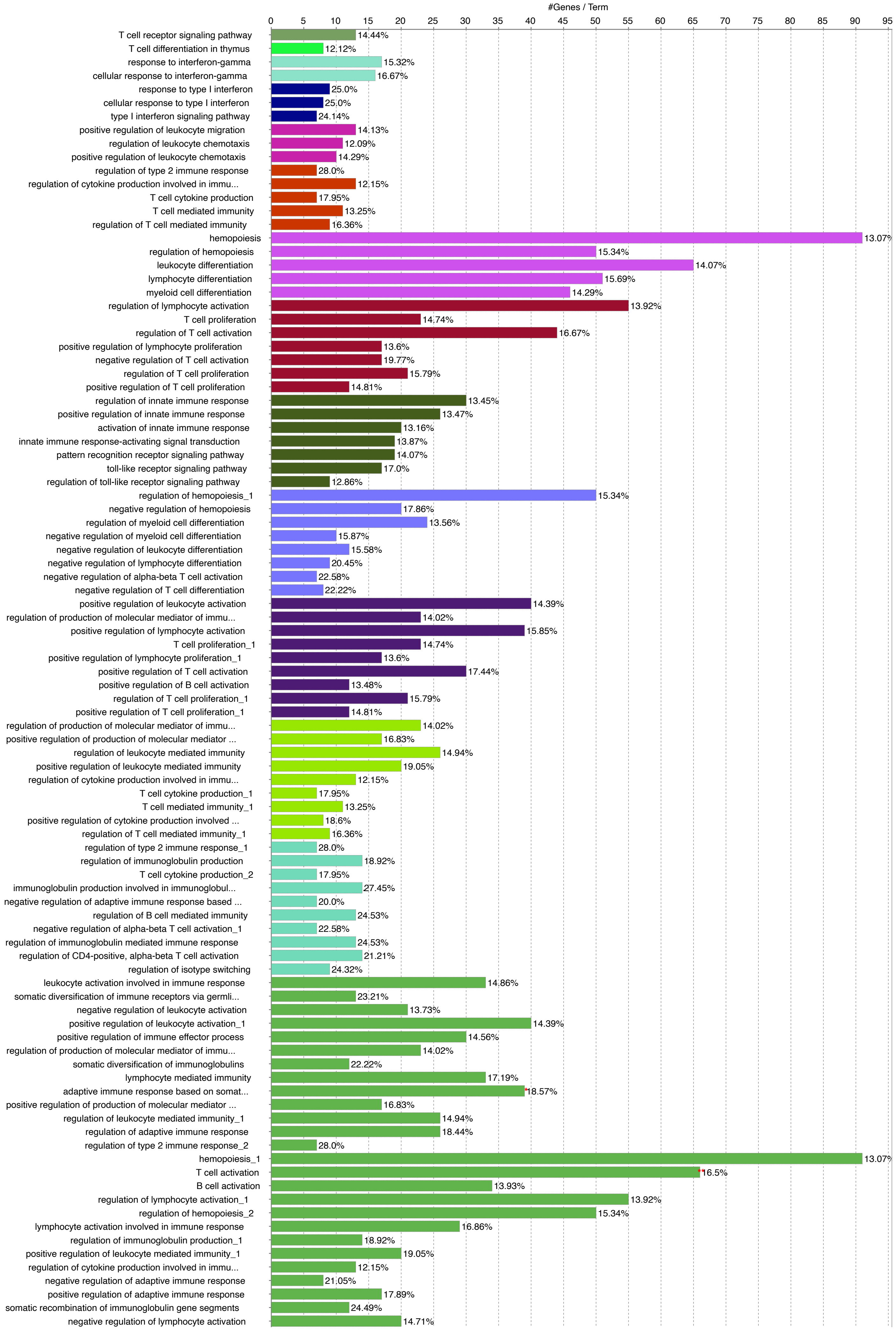

# Specific GO terms of immune processes of DEGs in Naïve_PMA/Ionomycin

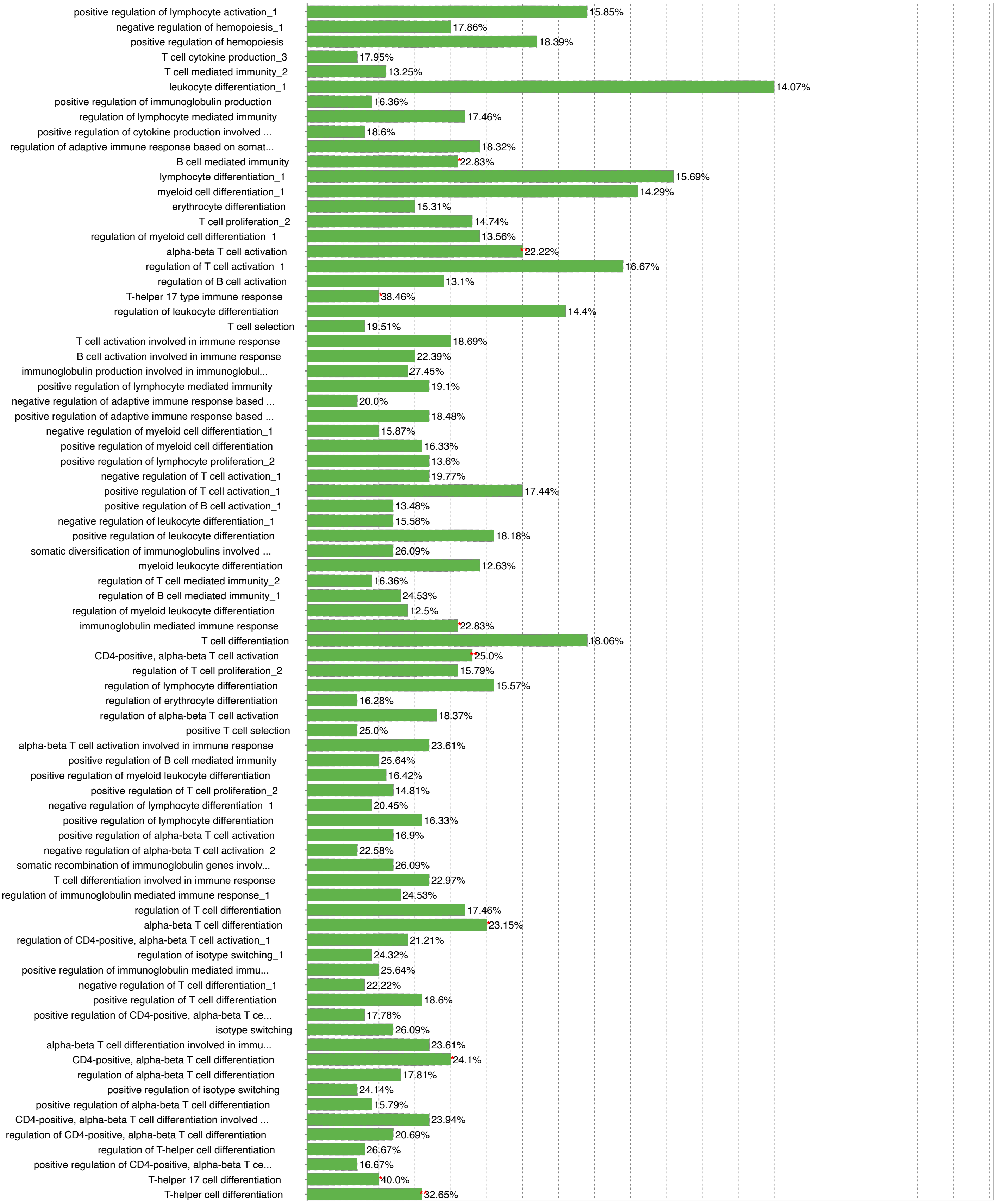

# Overview of immune processes of DEGs in Intermediate_PMA/Ionomycin

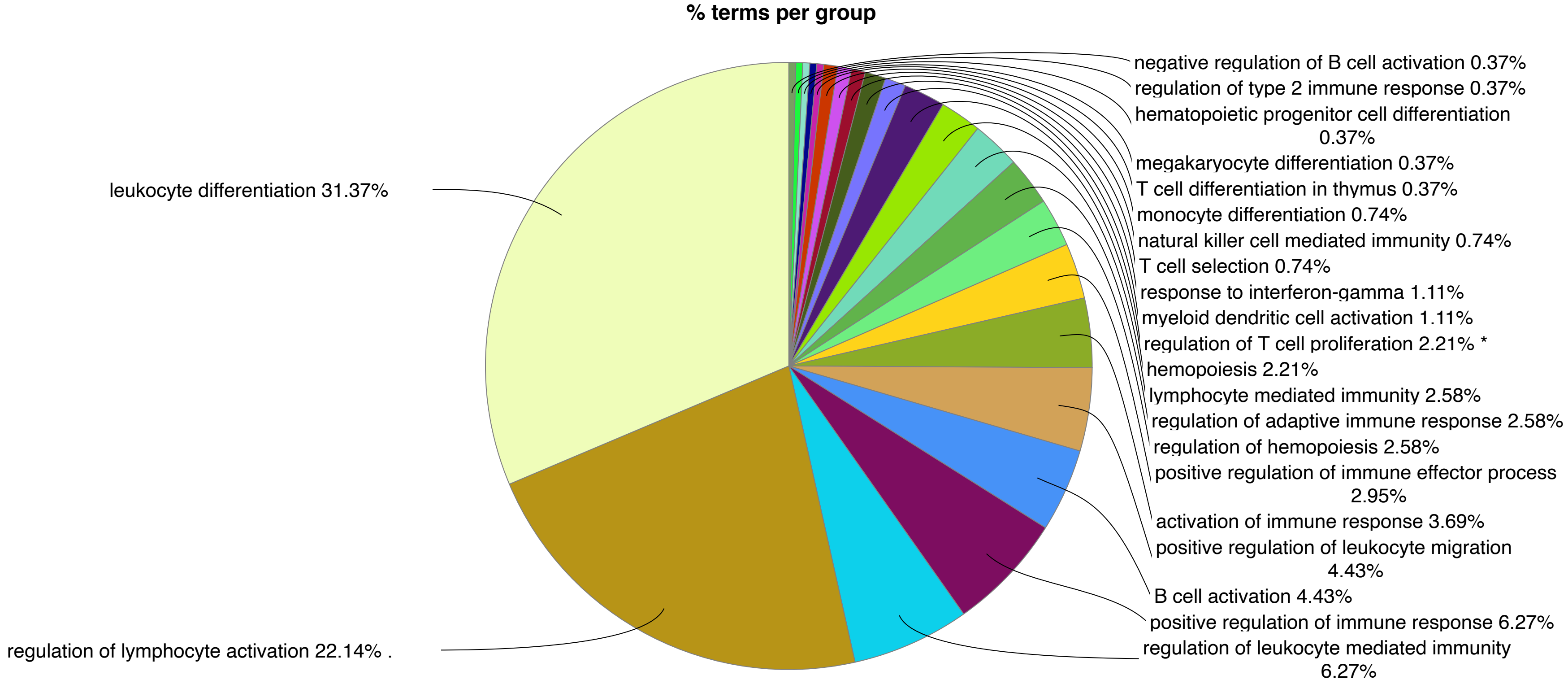

# Specific GO terms of immune processes of DEGs in Intermediate_PMA/Ionomycin

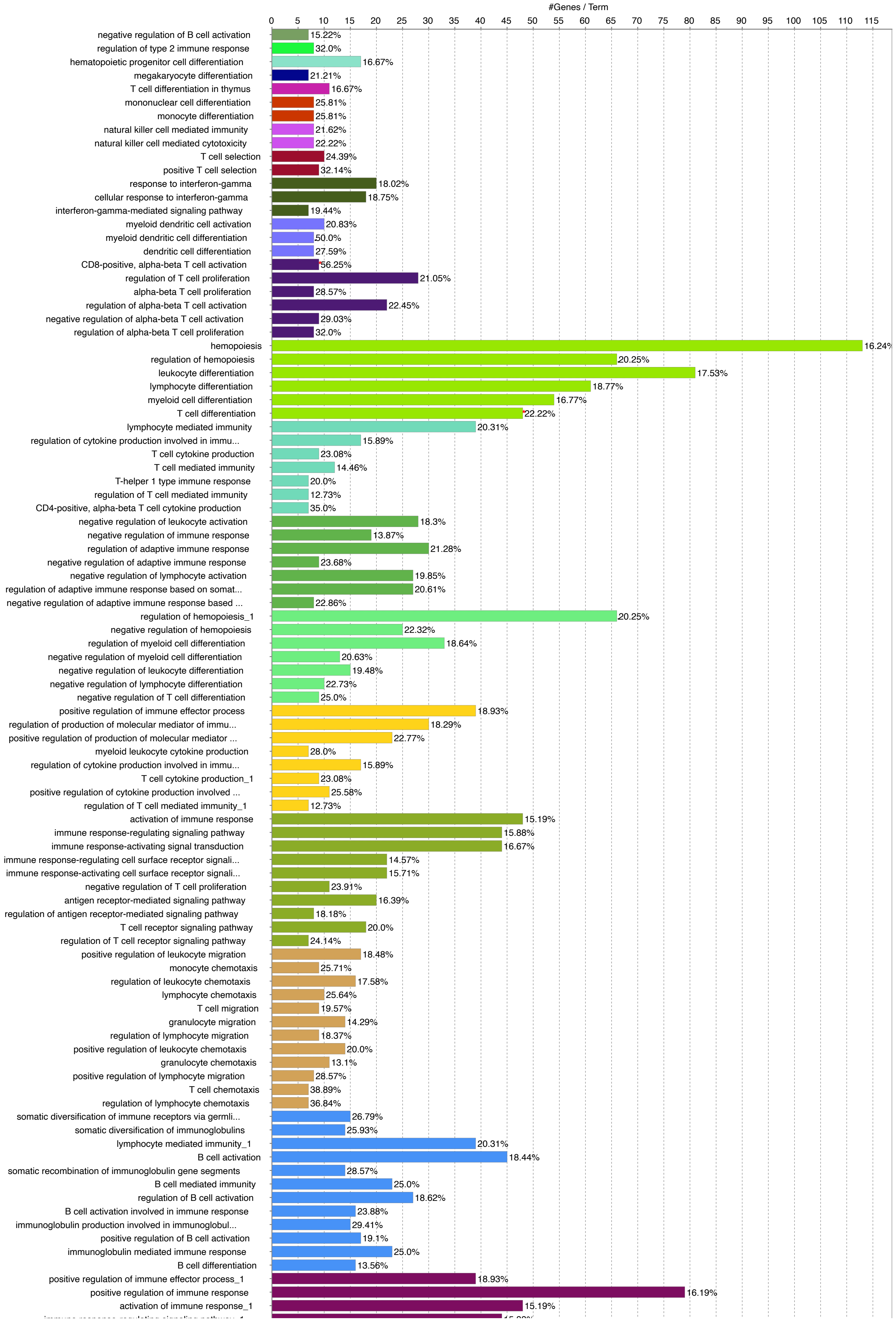

# Specific GO terms of immune processes of DEGs in Intermediate_PMA/Ionomycin

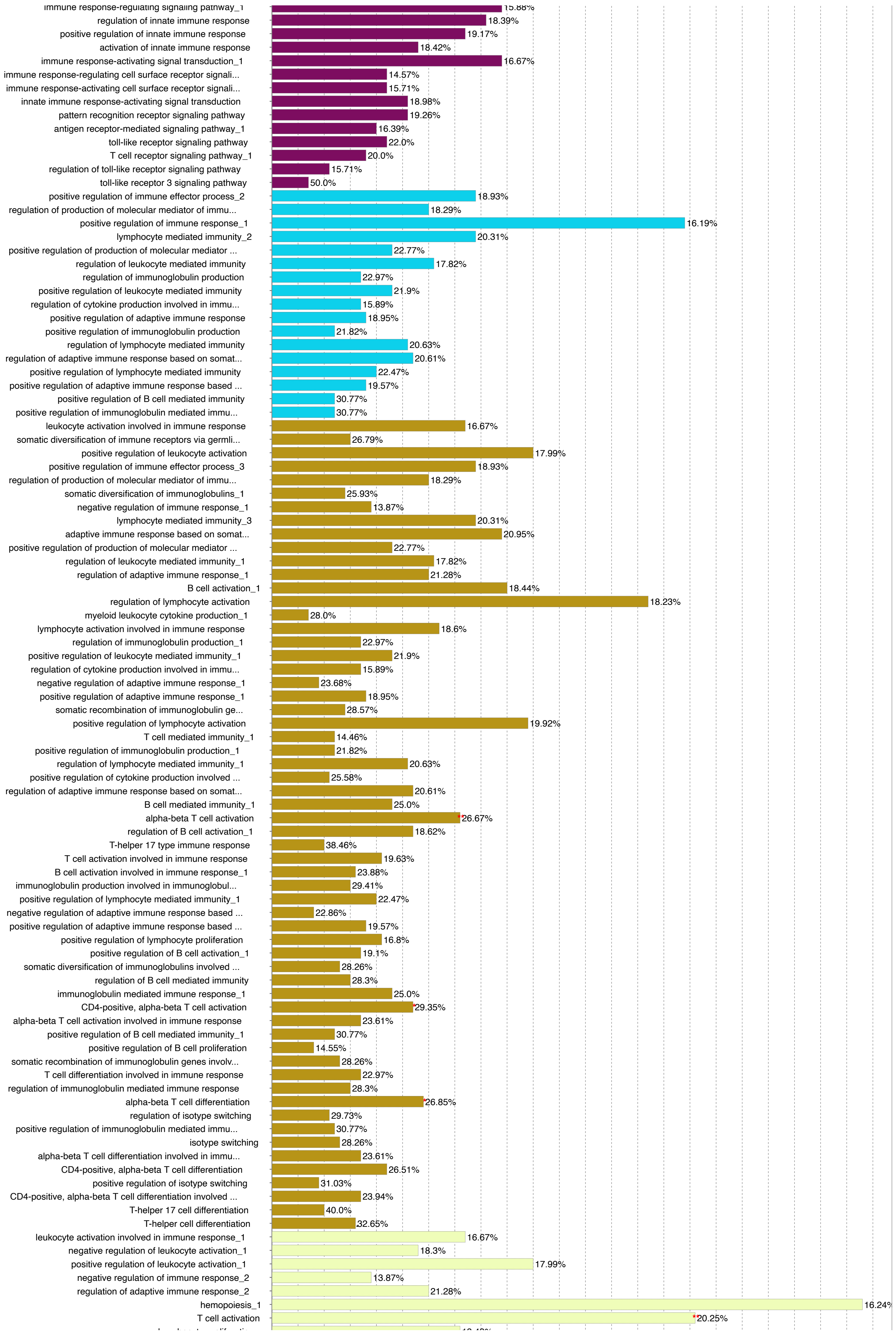

# Specific GO terms of immune processes of DEGs in Intermediate_PMA/Ionomycin

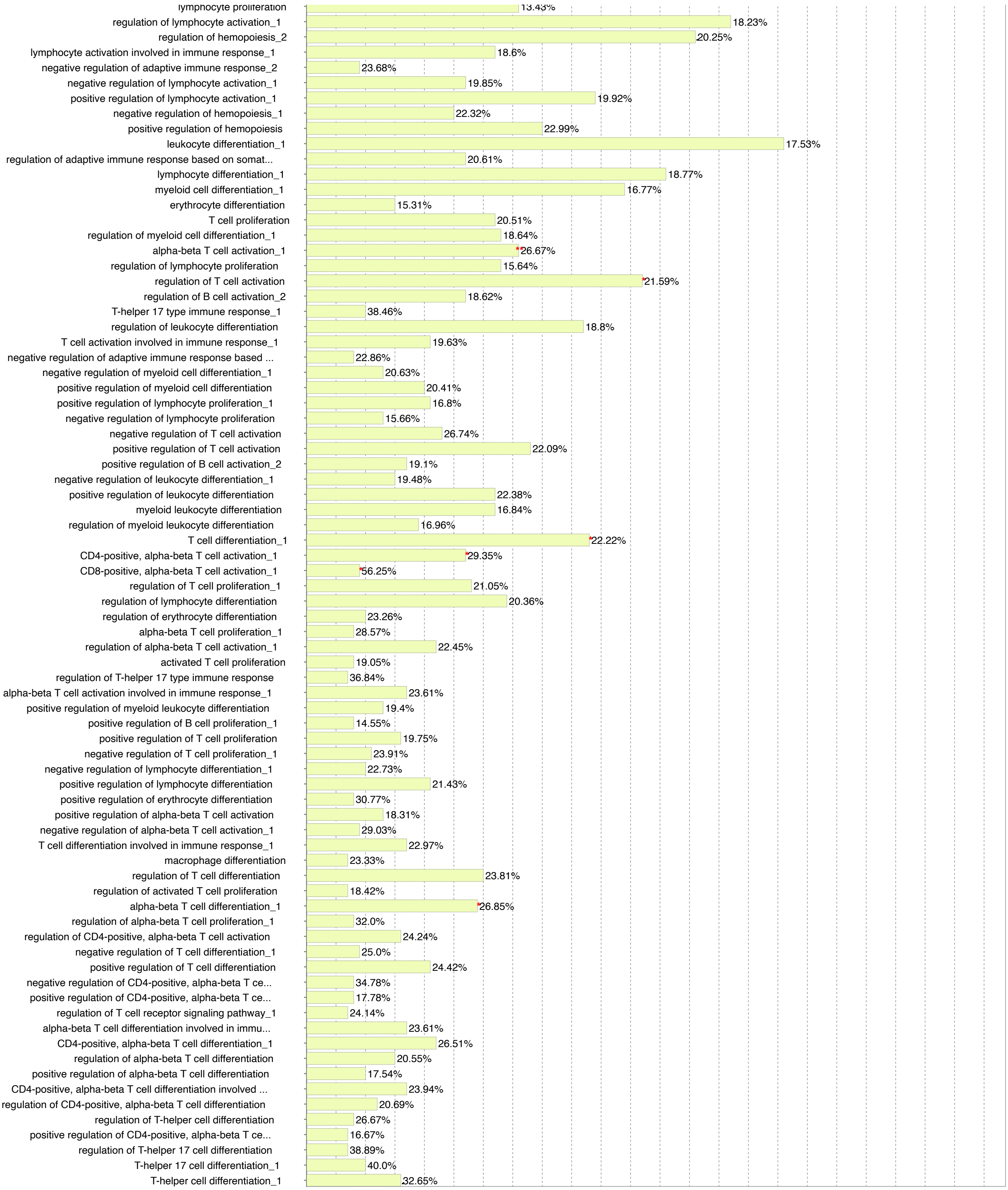

# Overview of immune processes of DEGs in Terminally_PMA/Ionomycin

**% terms per group**

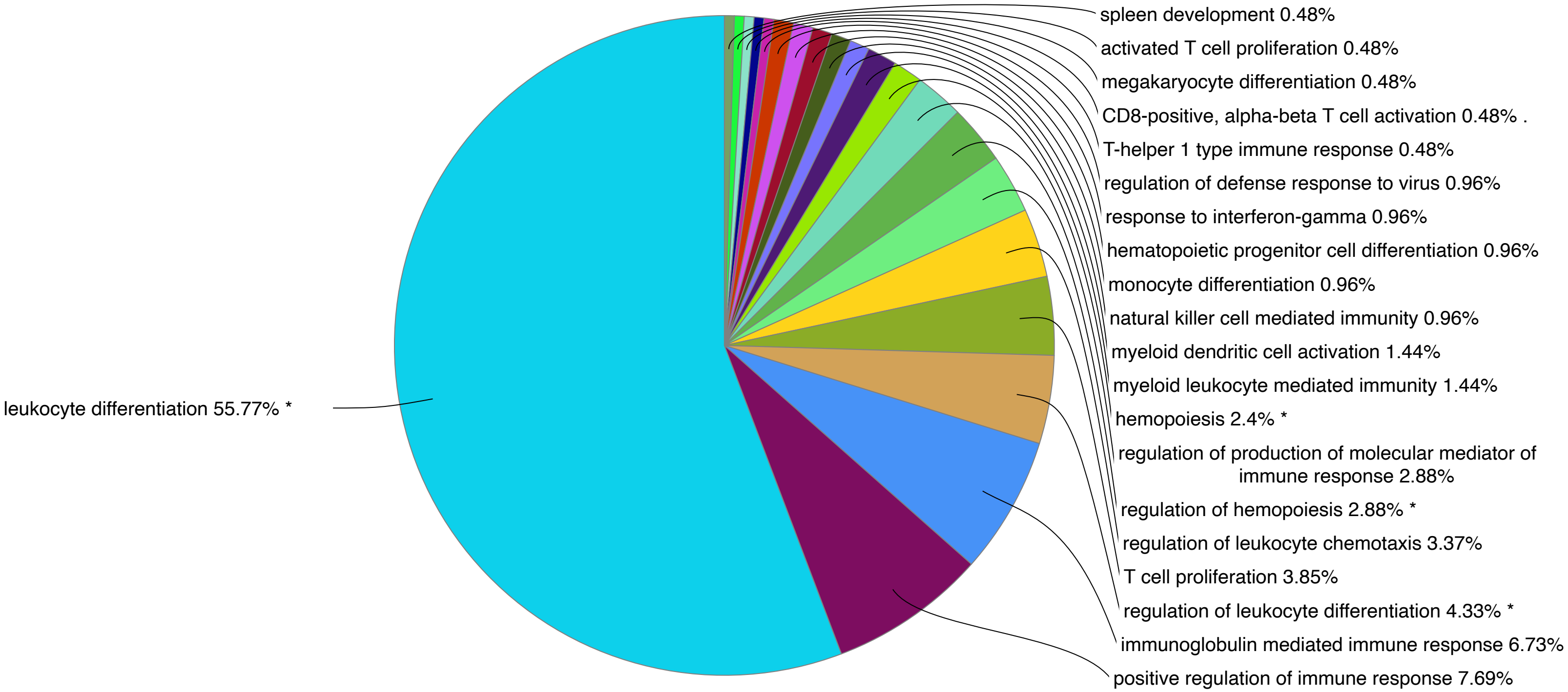

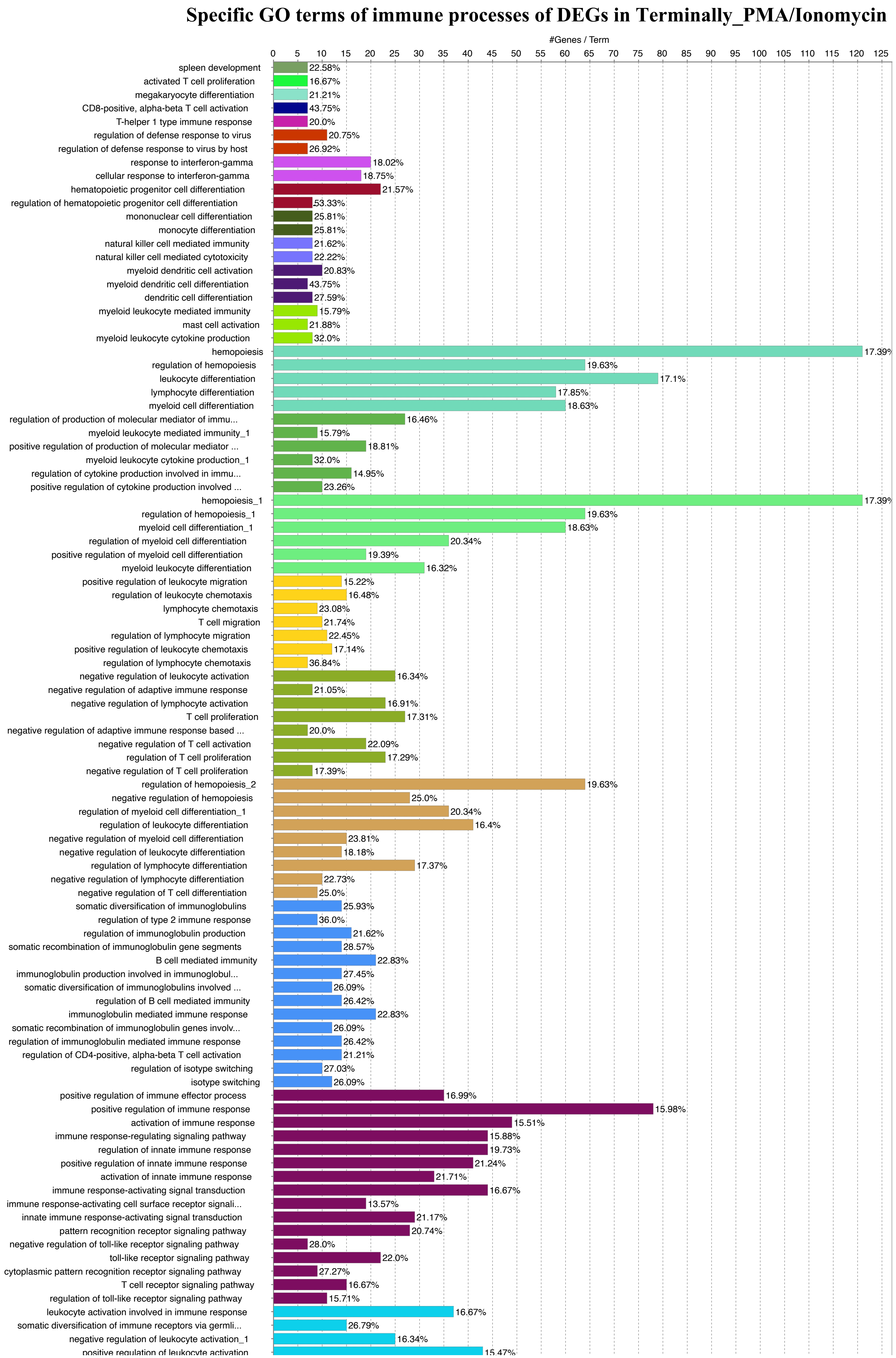

Specific GO terms of immune processes of DEGs in Terminally_PMA/Ionomycin
#Genes / Term
spleen development 22.58%
activated T cell proliferation 16.67%
megakaryocyte differentiation 21.21%
CD8-positive, alpha-beta T cell activation 43.75%
T-helper 1 type immune response 20.0%
regulation of defense response to virus 20.75%
regulation of defense response to virus by host 26.92%
response to interferon-gamma 18.02%
cellular response to interferon-gamma 18.75%
hematopoietic progenitor cell differentiation 21.57%
regulation of hematopoietic progenitor cell differentiation 53.33%
mononuclear cell differentiation 25.81%
monocyte differentiation 25.81%
natural killer cell mediated immunity 21.62%
natural killer cell mediated cytotoxicity 22.22%
myeloid dendritic cell activation 20.83%
myeloid dendritic cell differentiation 43.75%
dendritic cell differentiation 27.59%
myeloid leukocyte mediated immunity 15.79%
mast cell activation 21.88%
myeloid leukocyte cytokine production 32.0%
hemopoiesis 17.39%
regulation of hemopoiesis 19.63%
leukocyte differentiation 17.1%
lymphocyte differentiation 17.85%
myeloid cell differentiation 18.63%
regulation of production of molecular mediator of immu... 16.46%
myeloid leukocyte mediated immunity_1 15.79%
positive regulation of production of molecular mediator ... 18.81%
myeloid leukocyte cytokine production_1 32.0%
regulation of cytokine production involved in immu... 14.95%
positive regulation of cytokine production involved ... 23.26%
hemopoiesis_1 17.39%
regulation of hemopoiesis_1 19.63%
myeloid cell differentiation_1 18.63%
regulation of myeloid cell differentiation 20.34%
positive regulation of myeloid cell differentiation 19.39%
myeloid leukocyte differentiation 16.32%
positive regulation of leukocyte migration 15.22%
regulation of leukocyte chemotaxis 16.48%
lymphocyte chemotaxis 23.08%
T cell migration 21.74%
regulation of lymphocyte migration 22.45%
positive regulation of leukocyte chemotaxis 17.14%
regulation of lymphocyte chemotaxis 36.84%
negative regulation of leukocyte activation 16.34%
negative regulation of adaptive immune response 21.05%
negative regulation of lymphocyte activation 16.91%
T cell proliferation 17.31%
negative regulation of adaptive immune response based ... 20.0%
negative regulation of T cell activation 22.09%
regulation of T cell proliferation 17.29%
negative regulation of T cell proliferation 17.39%
regulation of hemopoiesis_2 19.63%
negative regulation of hemopoiesis 25.0%
regulation of myeloid cell differentiation_1 20.34%
regulation of leukocyte differentiation 16.4%
negative regulation of myeloid cell differentiation 23.81%
negative regulation of leukocyte differentiation 18.18%
regulation of lymphocyte differentiation 17.37%
negative regulation of lymphocyte differentiation 22.73%
negative regulation of T cell differentiation 25.0%
somatic diversification of immunoglobulins 25.93%
regulation of type 2 immune response 36.0%
regulation of immunoglobulin production 21.62%
somatic recombination of immunoglobulin gene segments 28.57%
B cell mediated immunity 22.83%
immunoglobulin production involved in immunoglobul... 27.45%
somatic diversification of immunoglobulins involved ... 26.09%
regulation of B cell mediated immunity 26.42%
immunoglobulin mediated immune response 22.83%
somatic recombination of immunoglobulin genes involv... 26.09%
regulation of immunoglobulin mediated immune response 26.42%
regulation of CD4-positive, alpha-beta T cell activation 21.21%
regulation of isotype switching 27.03%
isotype switching 26.09%
positive regulation of immune effector process 16.99%
positive regulation of immune response 15.98%
activation of immune response 15.51%
immune response-regulating signaling pathway 15.88%
regulation of innate immune response 19.73%
positive regulation of innate immune response 21.24%
activation of innate immune response 21.71%
immune response-activating signal transduction 16.67%
immune response-activating cell surface receptor signali... 13.57%
innate immune response-activating signal transduction 21.17%
pattern recognition receptor signaling pathway 20.74%
negative regulation of toll-like receptor signaling pathway 28.0%
toll-like receptor signaling pathway 22.0%
cytoplasmic pattern recognition receptor signaling pathway 27.27%
T cell receptor signaling pathway 16.67%
regulation of toll-like receptor signaling pathway 15.71%
leukocyte activation involved in immune response 16.67%
somatic diversification of immune receptors via germli... 26.79%
negative regulation of leukocyte activation_1 16.34%
positive regulation of leukocyte activation 15.47%

# Specific GO terms of immune processes of DEGs in Terminally_PMA/Ionomycin

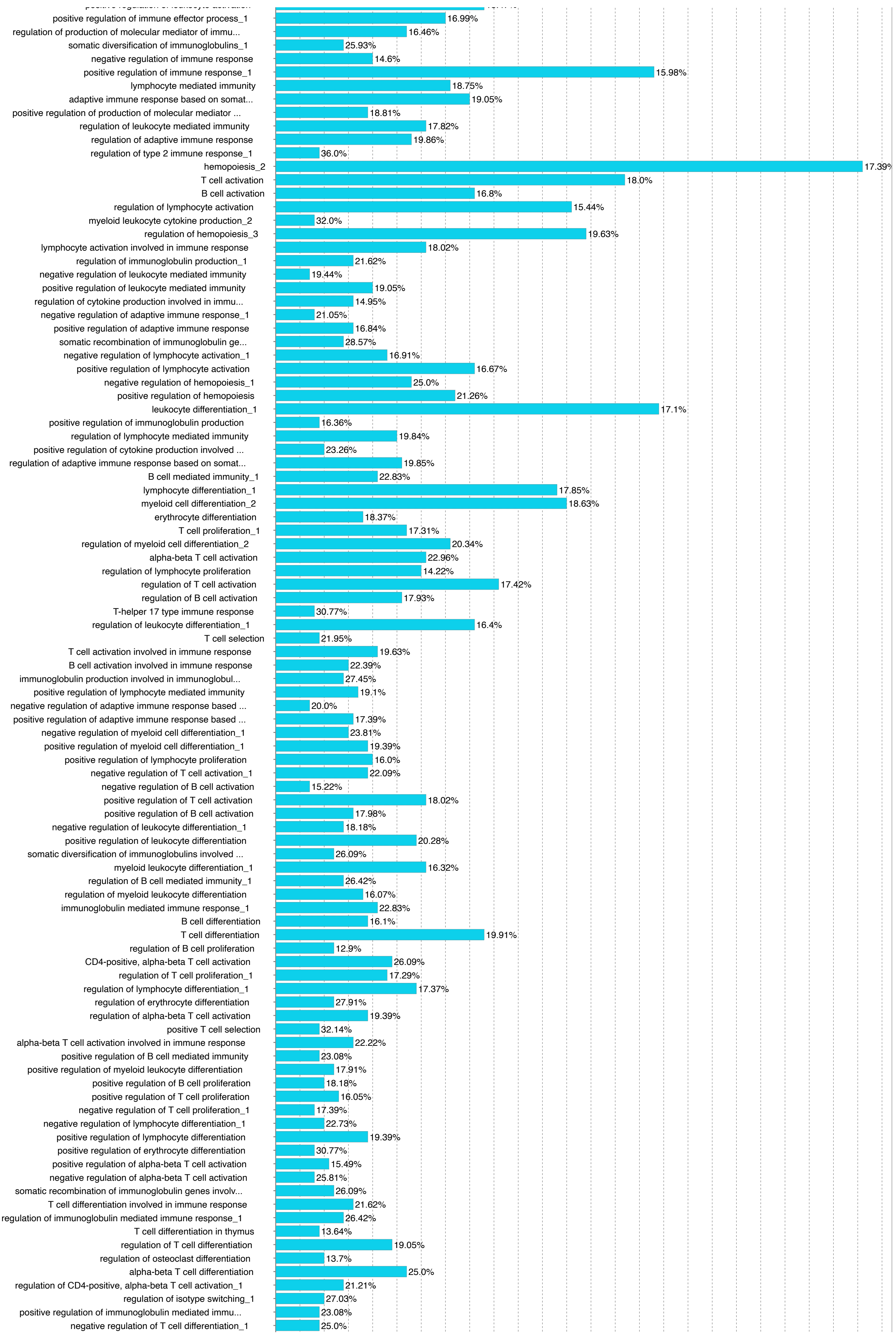

## Specific GO terms of immune processes of DEGs in Terminally_PMA/Ionomycin

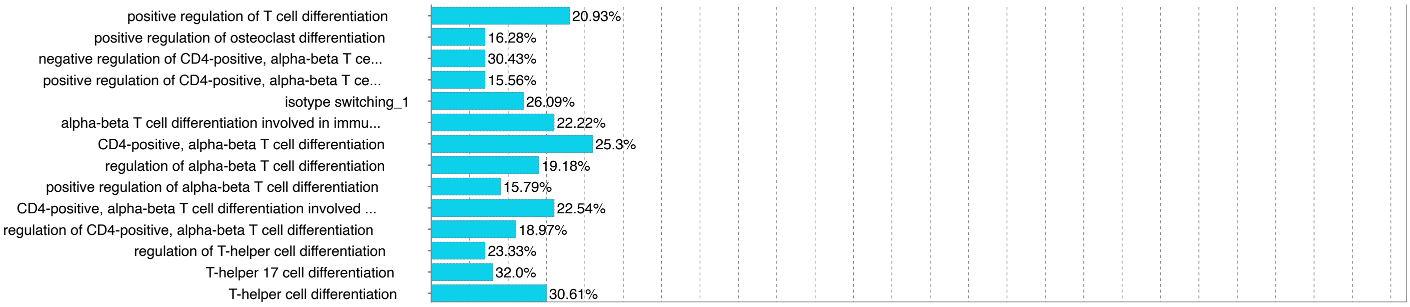

Supplement: Supplementary file 2 [file DataSheet_2.pdf]
